# A microbiome–metabolome signature associated with pediatric severe asthma

**DOI:** 10.1371/journal.pone.0358560

**Published:** 2026-09-18

**Authors:** Mélanie Briard, Blanche Guillon, Eric Venot, Marta Grauso, Christelle Hennequet-Antier, Aurélia Bruneau, François Fenaille, Florence Castelli, Muriel Thomas, Guillaume Lezmi, Maria Leite-de-Moraes, Karine Adel-Patient, Vinciane Saint-Criq

**Affiliations:** 1 Université Paris-Saclay, CEA, INRAE, UMR Département Médicaments et Technologies pour la Santé (DMTS)/SPI/Laboratoire d’Immuno-Allergie Alimentaire, Gif-sur-Yvette, France; 2 Université Paris-Saclay, INRAE, AgroParisTech, UMR1319 Micalis Institute, Jouy-en-Josas, France; 3 INRAE, Université Paris-Saclay, BioinfOmics, MIGALE Bioinformatics Facility, Jouy-en-Josas, France; 4 Université Paris-Saclay, INRAE, MaIAGE, 78350 Jouy-en-Josas, France; 5 Université Paris-Saclay, CEA, INRAE, UMR Département Médicaments et Technologies pour la Santé (DMTS)/SPI/Laboratoire innovations en spectrométrie de masse pour la santé, MetaboHUB, Gif-sur-Yvette, France; 6 AP-HP, Hôpital Necker-Enfants Malades, Service de Pneumologie et Allergologie Pédiatriques, Paris, France; 7 Université Paris Cité, Institut Necker Enfants Malades, Equipe Immunorégulation et Immunopathologie, Inserm UMR1151, CNRS, Paris, France; UAE University: United Arab Emirates University, UNITED ARAB EMIRATES

## Abstract

**Background:**

Severe asthma is a heterogeneous condition encompassing multiple phenotypes. Understanding lung-specific mechanisms in children with severe asthma may enable the development of more precise therapeutic strategies. We previously reported that immune components in bronchoalveolar lavages (BALs) differentiate children with severe asthma from non-asthmatic disease-controls and, frequent from non-frequent exacerbators, among children with severe asthma.

**Objective:**

To identify a local signature of severe asthma using complementary multi-omics analyses of BALs. A secondary objective was to evaluate whether bacterial taxa and metabolites discriminate severe asthma subtypes associated with distinct phenotypes or endotypes.

**Methods:**

BAL microbiome and metabolome were investigated in 20 children with severe asthma and 10 non-asthmatic children using 16S rRNA gene amplicon sequencing and liquid chromatography coupled to high-resolution mass spectrometry (LC-HRMS), respectively. Data were analysed separately and through integrative multi-omics approaches.

**Results:**

Compared with controls, BALs from children with severe asthma showed increased alpha-diversity, higher relative abundances of *Actinobacteriota*, *Streptococcus*, *Moraxella*, *Corynebacterium*, *Tropheryma*, and *Treponema*, and an altered polyamine pathway characterized by reduced arginine and increased spermine and spermidine levels. Integrated analyses revealed significant associations between *Streptococcus* and both spermine and spermidine. Independently, each dataset discriminated severe asthma phenotypes, notably exacerbation frequency and co-occurring atopic dermatitis. Unsupervised clustering of microbiome profiles identified four distinct clusters that may reflect severe asthma endotypes.

**Conclusions:**

This study identifies a distinct airway microbiome–metabolome signature associated with pediatric severe asthma. Enrichment of specific bacterial taxa, particularly *Streptococcus*, together with altered polyamine metabolic pathway, highlights microbial–metabolic interactions potentially involved in disease pathophysiology. The ability of microbiome and metabolome profiles to independently and jointly discriminate clinical phenotypes underscores the relevance of multi-omics approaches for diagnosis and follow-up of severe asthma. Our findings support the importance of airway-level profiling to improve mechanistic understanding of severe asthma and inform on future targeted therapeutic strategies.

## Introduction

Asthma is a chronic inflammatory airway disease marked by hyperresponsiveness to irritants, allergens, or viral infections (Global Initiative for Asthma. Global strategy for asthma management and prevention. www.ginasthma.org (2022)). Traditionally, asthma has been classified into T2-high (eosinophilic) and T2-low (non-eosinophilic) endotypes [1,2]. More recent studies, however, reveal involvement of additional pathways, including T1, T2, and T17 responses, underscoring immune complexity beyond a simple T2 paradigm [3–5]. In some individuals, asthma remains uncontrolled despite high-dose inhaled corticosteroids (ICS), additional controllers, correct medication use, and elimination of modifiable risk factors such as tobacco exposure, defining severe asthma (SA) [6]. SA is clinically heterogeneous and accounts for a disproportionate share of morbidity, healthcare use, and impaired quality of life [7–10]. Although biologics have emerged as promising therapies for SA, their optimal use requires understanding SA phenotypes (clinical traits), each of which results from different endotypes (underlying molecular mechanisms) [1,11]. Because many patients remain unresponsive and local–systemic signatures correlate poorly [3], deeper tissue-level characterization and biomarker discovery are needed.

Contrary to long-held assumptions, the lower respiratory tract harbours a dynamic microbial community that interacts with the host epithelial barrier and immune system. The lower airway microbiome influences immune homeostasis and airway inflammation [12] and differs from that of the upper airway [13]. An imbalance between symbiotic and pathogenic bacterial strains in the lung may lead to altered immune development and inappropriate inflammatory responses [14,15]. Indeed, altered lung microbiota composition has been associated with susceptibility to and/or severity of childhood respiratory diseases, including asthma [16–20]. Studies show that the bronchial and sputum microbiomes differ in asthmatic versus healthy adults, with higher abundance of *Actinobacteriota*, *Haemophilus*, *Moraxella*, *Staphylococcus*, and *Streptococcus* [21–24]. In contrast, data on the lower airway microbiota in children with asthma remain scarce; most pediatric studies have focused on the upper airway, though these studies suggest comparable microbial shifts [17–20]. One study investigated the upper and lower airway microbiomes in children with SA and found significantly different communities between the two locations, and a negative association between *Actinomyces* and transcripts related to inflammation [25].

Metabolites in bronchoalveolar lavage (BAL) fluid reflect the combined biochemical activities of microbes and host cells, providing more direct insight into airway physiology, inflammation and remodeling than microbial taxonomy alone [26,27]. Advances in untargeted and targeted metabolomic approaches, including liquid chromatography coupled to high-resolution mass spectrometry (LC–HRMS), have enabled detection of lipid mediators, amino acids, and microbial by-products in BALs, highlighting pathways central to immune responses and airway physiology [28]. To date, pediatric BAL metabolomics, particularly in children with SA, remains rare, limiting our understanding of how host–microbe interactions manifest at the functional level in diseased lung tissue. Indeed, attempts to identify biomarkers of childhood asthma using metabolomics, have focused primarily on blood, urine, and exhaled breath condensate samples [29]. We found only two studies investigating the BAL metabolome in childhood asthma [28,30], of which one did not compare its data to non-asthmatic controls [30]. The other study indicated that children with persistent wheezing, but not SA, had higher abundances of choline, oleamide, butyrylcarnitine, palmitoylethanolamide, and various phosphatidylcholines, suggesting alterations in local metabolism [28].

Recently, we showed that a large set of immune components in BAL fluids differentiated children with severe asthma (SA) from disease-control subjects [3], and that distinct immune signatures further discriminated frequent from non-frequent exacerbators among children with SA [31]. Given the scarcity of studies of the BAL microbiome and metabolome in pediatric SA, we hypothesized that integrative microbiome–metabolome profiling would provide new insights into the pathophysiology of pediatric SA. To test this hypothesis, we used 16S rRNA gene amplicon sequencing and untargeted LC-HRMS metabolomics to identify microbial taxa and metabolites, respectively, associated with SA, and exacerbation frequency or atopic dermatitis (AD). We then applied the Data Integration Analysis for Biomarker discovery using Latent cOmponents (DIABLO [32]) method to investigate the link between taxa and metabolites. Unsupervised clustering of these datasets further revealed SA subgroups, which may correspond to distinct SA endotypes. This highlights the need for validation in larger patient cohorts and for assessing their association with individual trajectories and response to treatment.

## Materials and Methods

### Study population and sample processing

Twenty school-aged children with severe asthma (SA) were included. Patients were mainly from the Ile-de-France region, and followed in the department of paediatric pulmonology and allergy at Necker Hospital (Paris, France). Ten children, with chronic respiratory disorders unrelated to asthma who required endoscopy, were also recruited as age-matched disease-control subjects (hereafter referred to as non-asthmatic, NA). NA subjects had chronic respiratory disorders, including ciliary dyskinesia (n = 2), viral pulmonary sequelae (n = 3), or non-cystic fibrosis bronchiectasis (n = 5). Clinical data, patient characteristics and ethical statements are described in our previous studies [3,31,33] and summarized in S1 Table. Patients included in this study were from the CLASSE (Cellules Lymphoïdes Innées dans l’ASthme Sévère de l’Enfant) cohort. They were recruited in the department of Paediatric Pulmonology and Allergy at Necker Hospital (Paris, France) between 05/07/2016 and 14/06/2018. Written and oral information about the study was provided to patients and their parents/guardians, and institutional ethical approval (ref 2016-03-07 RNI) and written informed consent from parents/guardians were obtained.

The BAL microbiota and metabolite datasets underlying this study have been published as a data paper [34], which provides full details of BAL fluid sampling, measurements, and data pre-treatment and curation. The datasets generated and analyzed in the current study are available at data.inrae.fr (metabolites [35]; microbiota: [36]). Here, we summarize the key aspects relevant to the present analysis.

### Microbiota analyses

DNA extraction from BAL fluids dry pellets was performed using the QIAamp Power Fecal DNA kit and following the manufacturer’s instructions (Qiagen; Courtaboeuf, France). Extraction blanks were processed alongside all BAL samples by performing the complete DNA extraction protocol on tubes containing no biological material. These blanks served as negative controls to monitor for potential contamination introduced during extraction or laboratory handling, or by reagents.

BAL bacterial load was measured by real-time qPCR on BAL DNA samples diluted 1/10 using the BACT1369F forward primer (5’-CGGTGAATACGTTCCCGG-3’), the PROK1492R reverse primer (5’-GGCTACCTTGTTACGACTT-3’) and the TM1389F probe (5’-6-FAM-CTTGTACACACCGCCCGTC-3’), as previously described [37,38] (amplification efficiency 93%).

Data processing of bacterial 16S rRNA gene amplicon sequences and statistical analyses were performed in RStudio (R version 4.4.1). Raw ASV counts, taxonomy, and sample metadata were imported into phyloseq (version 1.50.0). To reduce sparsity and low-abundance noise, ASVs detected in fewer than 10% of samples and with relative abundance below 0.01% were excluded from downstream analyses. For diversity and ordination, feature tables were total-sum scaled (TSS) to relative abundance. Alpha diversity indices (observed richness, Chao1, Shannon, inverse Simpson) were computed with estimate_richness and group comparisons used the Wilcoxon rank-sum test. Beta diversity was assessed using Bray–Curtis dissimilarities calculated from total-sum scaled ASV counts. Ordinations were performed by principal coordinates analysis (PCoA) using the ordinate function in the phyloseq R package. Group differences in microbial community composition were tested using permutational multivariate analysis of variance (PERMANOVA; adonis2 function in vegan v2.7-2) with 999 permutations. Homogeneity of group dispersions was evaluated prior to PERMANOVA using the betadisper and permutest functions (vegan). Graphical representations of ordinations were generated in R with ggplot2, following the code structure and workflow described by van Beveren *et al.* [39].

Differential abundance analyses were performed using multiple complementary methods. For compositional approaches, ALDEx2 (v1.38.0; centered log-ratio transformation denom = “all”, 250 Monte Carlo instances, Welch’s t-test, effect sizes with 95% CI) and ANCOM-BC2 (v2.8.0; fixed-effects model fix_formula=”asthma”, prv_cut = 0.1, lib_cut = 1000, structural zeros struc_zero = TRUE, neg_lb = TRUE, global test global = TRUE, EM control tol = 1e-5, max_iter = 100, BH-adjusted p-values, alpha = 0.05) were applied. Count-based methods included DESeq2 (v1.46.0; median-of-ratios size-factor normalization, Wald test, parametric dispersion fit, log-fold change shrinkage via apeglm) and edgeR (v4.4.0; TMM normalization, negative-binomial GLM, quasi-likelihood F-test). Zero-inflated modelling was performed with metagenomeSeq (v1.43.0; CSS normalization percentile = 0.5, zero-inflated Gaussian model fitZig, BH-adjusted p-values). Multivariable linear modelling was performed with MaAsLin2 (v1.15.1; prevalence ≥10% min_prevalence = 0.1, no minimum abundance filter, log transform, linear model with fixed effects for asthma). Finally, LinDA (MicrobiomeStat v1.2; CLR transform, pseudo-count = 0.5, prevalence filter 10% prev.filter = 0.1, outlier trimming is.winsor = TRUE, outlier.pct = 0.03, adaptive bias correction adaptive = TRUE, BH-adjusted p-values, alpha = 0.05) was applied. Multiple testing correction was performed with the Benjamini–Hochberg method, and amplicon sequence variants were considered differentially abundant when the adjusted p-values were less than 0.05.

To balance sensitivity and false discovery risk in exploratory analyses, we used an adjusted p-value cutoff of 0.20 (rather than the conventional 0.05) in the initial candidate selection step. This liberal threshold was deliberately chosen to maximize detection of potential signals across diverse statistical frameworks (ALDEx2, ANCOM-BC2, DESeq2, edgeR, MaAsLin2, MetagenomeSeq, and LinDA), each of which may capture distinct aspects of the data due to differences in assumptions, normalization, or modeling approaches. By requiring consensus (taxon adjusted p-value < 0.20 in at least three methods at the ASV level or two methods at the genus level), we mitigated the risk of method-specific false positives and ensured robustness in downstream follow-up investigations. Final candidate taxa were those with reproducible signals across multiple independent analyses, reducing bias toward any single method.

Differential abundance analysis using DESeq2 was also performed to identify potential microbial signatures in SA children classified as frequent exacerbators (FE, n severe exacerbations ≥ 3 in the previous year), compared with non-frequent exacerbators (nFE, n severe exacerbations = 1–2 in the previous year) and with children with AD.

Unsupervised clustering of microbial communities was performed using hierarchical clustering based on Bray–Curtis dissimilarities. First, ASVs were ordered by overall abundance, and a Bray–Curtis distance matrix was computed from the relative abundance table using the vegdist function (vegan R package). Hierarchical clustering was then conducted with the hclust function using average linkage. To determine the optimal number of clusters, we evaluated partition quality for 2–10 clusters using two independent validity metrics: the Calinski–Harabasz index and the average silhouette width, as implemented in the cluster.stats function (fpc package v2.2-13). The number of clusters yielding the highest values of these indices was selected as the most appropriate solution (n = 4 clusters). To compare diversity across unsupervised clusters, linear models were fitted for each metric (“Observed”, “Chao1”, “Shannon”, “InvSimpson”) with cluster assignment as the explanatory variable. ANOVA was used to test for statistically significant differences among clusters. Model assumptions were checked via residual plots, and results were considered significant at p < 0.05. Cluster assignments were also visualized on PCoA ordinations of Bray–Curtis dissimilarities.

### Metabolome analyses

Peak areas of the 88 annotated metabolites from LC-HRMS analysis of BAL samples (described in [34] with the dataset available in [35]) were log10-transformed. Group comparisons between patients with SA and NA controls were performed using the Wilcoxon rank-sum test for each metabolite feature. Class discrimination was further assessed using partial least squares discriminant analysis (PLS-DA) with the mixOmics (version 6.30.0) R (version 4.4.3) package, which used two latent components. Model performance was evaluated using five-fold cross-validation repeated ten times, and the balanced error rate (BER) was computed as a measure of classification accuracy. Model significance was tested via 100 permutation analyses, in which class labels were randomly reassigned and BER values recalculated for each iteration. Receiver operating characteristic (ROC) curves were generated from the PLS-DA component scores, and the area under the curve (AUC) was computed for each component and their mean. Variable Importance in Projection (VIP) scores were extracted from the PLS-DA model, averaged across the first two components, and used to rank metabolites according to their contribution to class separation. Metabolites with a mean VIP score > 1 and a nominal p-value < 0.05 were considered the most discriminant features.

Pathway analysis (integrating pathway enrichment and pathway topology analyses) was also performed on the peak intensity table of annotated metabolites using KEGG ID and MetaboAnalyst 6.0 [40], and using the Small Molecule Pathway Database (SMPDB) as a metabolite set library.

### Multi-omics analyses

We applied the DIABLO (Data Integration Analysis for Biomarker discovery using Latent cOmponents) approach from the mixOmics R package to integrate microbiota and metabolite data obtained from 20 SA patients and 10 NA disease controls. Prior to integration, ASV tables were transformed to relative abundances and subjected to arcsine square root transformation, while metabolite intensities were log-transformed as log(intensity + 1). The DIABLO model was constructed using pre-selected ASVs (adjusted p-value < 0.2 in at least 3 differential abundance analysis methods for ASVs) and metabolites (p-value <0.05 and VIP score >1 for metabolites), using block.splsda() with two latent components. Fifteen ASVs (out of 338) and 17 annotated metabolites (out of 88) were used to build the DIABLO model. Classification accuracy was further assessed using confusion matrices that compared predicted and observed class labels. Variable contributions to the first component were examined using loading values, and graphical representations of sample separation (plotIndiv) and variable importance (plotVar) were generated to visualize relationships between selected microbiota and metabolite features.

## Results

### Study population

BAL microbiota and metabolites from 20 severe asthmatic (SA) and 10 disease control (NA) children were studied. Demographic and clinical characteristics of this cohort were previously published elsewhere [3,31] (S1 Table). Briefly, significant differences associated with severe asthma were found in the doses of ICS received, post BD-FEV1/FVC values, blood eosinophil counts, BAL cell densities, particularly neutrophils and lymphocytes, and BAL cytokine/growth factor concentrations. No differences were observed for age, biological sex, or BMI.

### General characteristics of the BAL microbiota and metabolites

**Fig 1:**
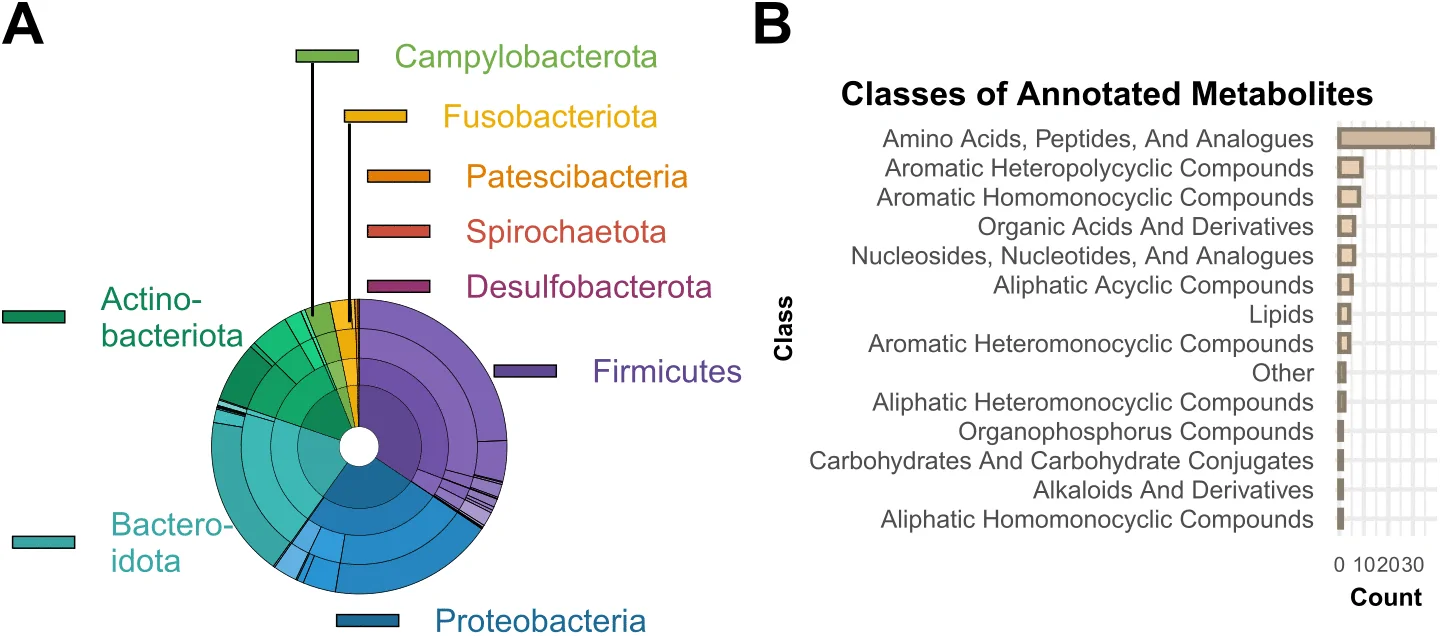
Bacterial taxa and metabolic classes of metabolites found in BAL from children, regardless of asthmatic status (n = 30). A. Circular hierarchical diagram illustrating the taxonomic composition of the airway microbiota. Each concentric ring represents a different taxonomic level, from phylum (inner ring) to family (outer ring). Major bacterial groups are color-coded by phylum. B. Bar plot showing the distribution of annotated metabolites across different classes. The x-axis represents the number of annotated metabolites in each chemical class, indicated on the y-axis.

The BAL microbiota was dominated by members of the phyla *Firmicutes*, *Proteobacteria*, *Bacteroidota*, and *Actinobacteriota*, which together accounted for the majority of the detected taxa. Less abundant phyla included *Campylobacterota*, *Fusobacteriota*, *Patescibacteria*, *Spirochaetota*, and *Desulfobacterota* (Fig 1A). Testing the association between demographical and clinical data and overall microbiota composition, independently of the severe asthma status revealed that although no covariates retained significance after false discovery rate (FDR) correction in PERMANOVA, several exposures and biomarkers—namely IgA, passive smoking, gestational smoking, anti-mite bedding, damp and mouldy housing, and blood lymphocyte and leukocyte counts—showed nominal associations before adjustment (S2 Table). Unexpectedly, neither age nor positive BAL virology was associated with variation in microbiota composition, despite their reported influence in previous studies [39,41]. Thus, these patterns suggest potential links between domestic exposure and BAL community structure, but the effects were not robust to correction for multiple comparisons and should be interpreted cautiously.

Unsupervised LC-HRMS allowed measuring the abundances of 88 metabolites in BAL samples [34]. These metabolites mainly belonged to the amino acids, peptides, organic acids, alkaloids, aromatic compounds, carbohydrates, nucleosides/nucleotides, lipids, and aliphatic chemical classes (Fig 1B). Correlation analyses between clinical/demographic characteristics and metabolic pathways, independent of asthmatic status, showed no association of pathways with age, BMI, or biological sex. In contrast, weak but significant associations were observed between lung bacterial infection and the arachidonic metabolism (ρ = –0.41, p = 0.025), lysine degradation (ρ = 0.37, p = 0.032), and inositol phosphate metabolism (ρ = 0.36, p = 0.048) pathways (S3 Table). At the metabolite level, most associations were found between amino acid and peptide derivatives, nucleotide metabolism intermediates, or lipid-derived inflammatory mediators and lung bacterial or viral infections (14 of 19 significant correlations, S4 Table).

### Identification of bacterial and metabolic biomarkers associated with severe asthma status

**Fig 2:**
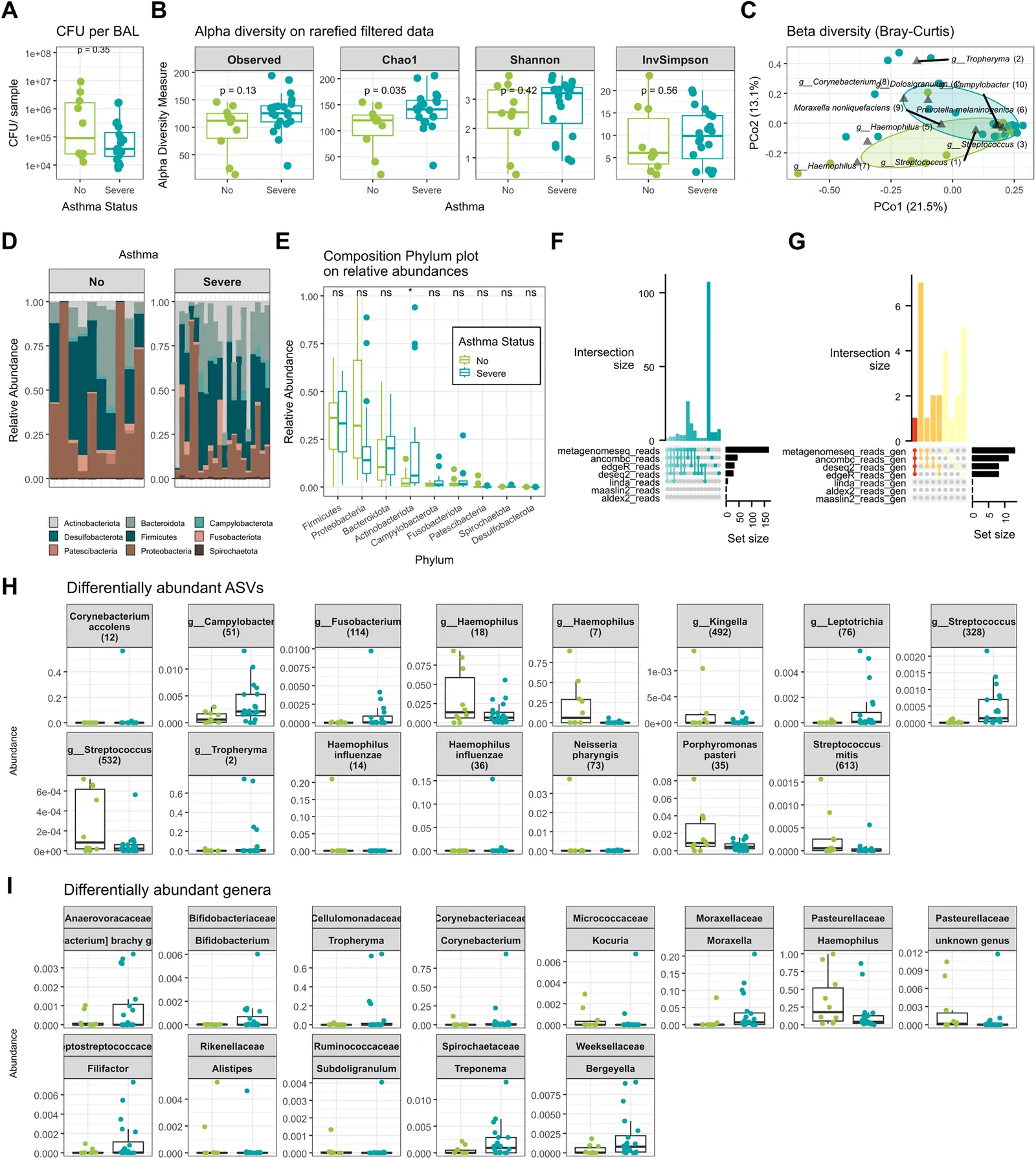
Microbiota composition and diversity, and microbial signatures in BAL from SA (blue) compared to NA (green) children. A. BAL bacterial load as a function of SA status, as measured by in-house TaqMan qPCR. B. Alpha diversity measures (observed ASVs, Chao1 richness estimator, Shannon diversity index, and inverse Simpson index) for the two groups. C. Principal coordinates analysis (PCoA) of Bray–Curtis dissimilarity, showing beta diversity patterns between NA and SA samples. Points represent individual samples, coloured by asthma status. Ellipses indicate the dispersion of samples within each group, corresponding to one standard deviation from the group centroid in the multivariate space. The top 10 highest ranking ASVs are visualized as triangles. D. Stacked bar plots showing the relative abundance of bacterial phyla in BAL samples from NA children (“no”, left panel) and those from SA children (“severe”, right panel). E. Boxplots of relative abundances at the phylum level, comparing BAL from NA to SA children. p-values from Wilcoxon rank-sum tests are shown above each phylum. F. UpSet plot showing the number of shared differentially abundant ASVs determined by multiple differential abundance analysis methods. Vertical bars indicate the number of ASVs included in the intersection of the methods. The colour underneath denotes the number of methods in the intersection. G. UpSet plot showing the number of shared differentially abundant genera determined by multiple differential abundance analysis methods. Vertical bars indicate the number of genera included in the intersection of the methods. The colour underneath denotes the number of methods in the intersection. H. Differentially abundant ASVs between BAL from SA (blue) and NA (green) children, identified by 3 or more methods. I. Differentially abundant genera in BAL between SA (blue) and NA (green) children, identified by 2 or more methods.

Total bacterial load was comparable in the BALs from children with SA and those without asthma (Fig 2A). Analysis of BALs microbiota using 16S rRNA gene amplicon sequencing revealed that alpha diversity differed between groups, with richness estimates (Observed ASVs, Chao1) higher in severe asthma (Observed p = 0.13; Chao1 p = 0.035), whereas diversity indices that account for evenness (Shannon, Inverse Simpson) did not show notable differences (Fig 2B). Beta diversity analysis using Bray–Curtis dissimilarity demonstrated a slight overlap among groups, and PERMANOVA based on Bray–Curtis dissimilarities revealed a statistically significant difference in overall microbiota composition between SA and NA children (R² = 0.0735, F = 2.22, p = 0.009). This result indicates that asthma status explained approximately 7% of the variation in community structure (Fig 2C). Dispersion did not differ significantly among groups (betadisper, p = 0.879), indicating that PERMANOVA results reflect centroid differences rather than heterogeneity of spread. Comparison of alpha and beta diversity indices between groups, using non-rarefied and/or non-filtered data, yielded similar results (S1 Fig.). Overall, phylum-level profiles were similar between the two groups, with *Firmicutes*, *Proteobacteria*, *Bacteroidota*, and *Actinobacteriota* being predominant in both groups (Fig 2D). However, *Actinobacteriota* showed a nominally higher relative abundance in SA individuals (p = 0.044; Fig 2E).

We then used metagenomeSeq, ANCOM-BC2, DESeq2, linDA, edgeR, maaslin2 and ALDEx2 to identify taxa with significantly different abundances between SA and NA BAL samples. Maaslin2, linDA, and ALDEx2 analyses did not reveal any significant differences in ASVs or genera between SA and NA after correction for multiple testing. However, metagenomeSeq, ANCOM-BC2, DESeq2, and edgeR identified 92, 14, 15, and 9 ASVs, and 8, 4, 6, and 2 genera, respectively, that showed significantly different abundances between SA and NA samples (adj-p-value <0.05, S2 Fig). To increase sensitivity in detecting potential biological signals across the complementary differential abundance methods employed and to select candidate taxa for follow-up investigations, we then used an adjusted p-value cut-off of 0.2 for all differential abundance analysis methods. We identified 15 ASVs (Fig 2 F,G) and 13 genera (Fig 2 H,I) significantly different between SA and NA samples by at least 3 and 2 methods, respectively. The multi-affiliated *Streptococcus* (ASV 328) was significantly increased in SA across 5 methods. *C. accolens* (12), *Campylobacter* (51), *Fusobacterium* (114), *Leptotrichia* (76), *Tropheryma* (2), and *H. influenzae* (36), were found significantly increased, whereas *Haemophilus* species (ASVs 18, 7, and 36), *Kingella* (492), *Streptococcus* (532), *N. pharyngis* (73), *P. pasteri*, and *S. mitis* were found significantly decreased in SA compared to NA (Fig 2F). At the genus level, *Eubacterium* brachy group, *Bifidobacterium*, *Tropheryma*, *Corynebacterium*, *Moraxella*, *Filifactor*, *Subdoligranulum*, *Treponema*, and *Bergeyella* were enriched in SA individuals, whereas *Kocuria*, *Haemophilus*, an unknown genus in the *Pasteurellaceae* family, and *Alistipes* were increased in NA children (Fig 2I).

**Fig 3:**
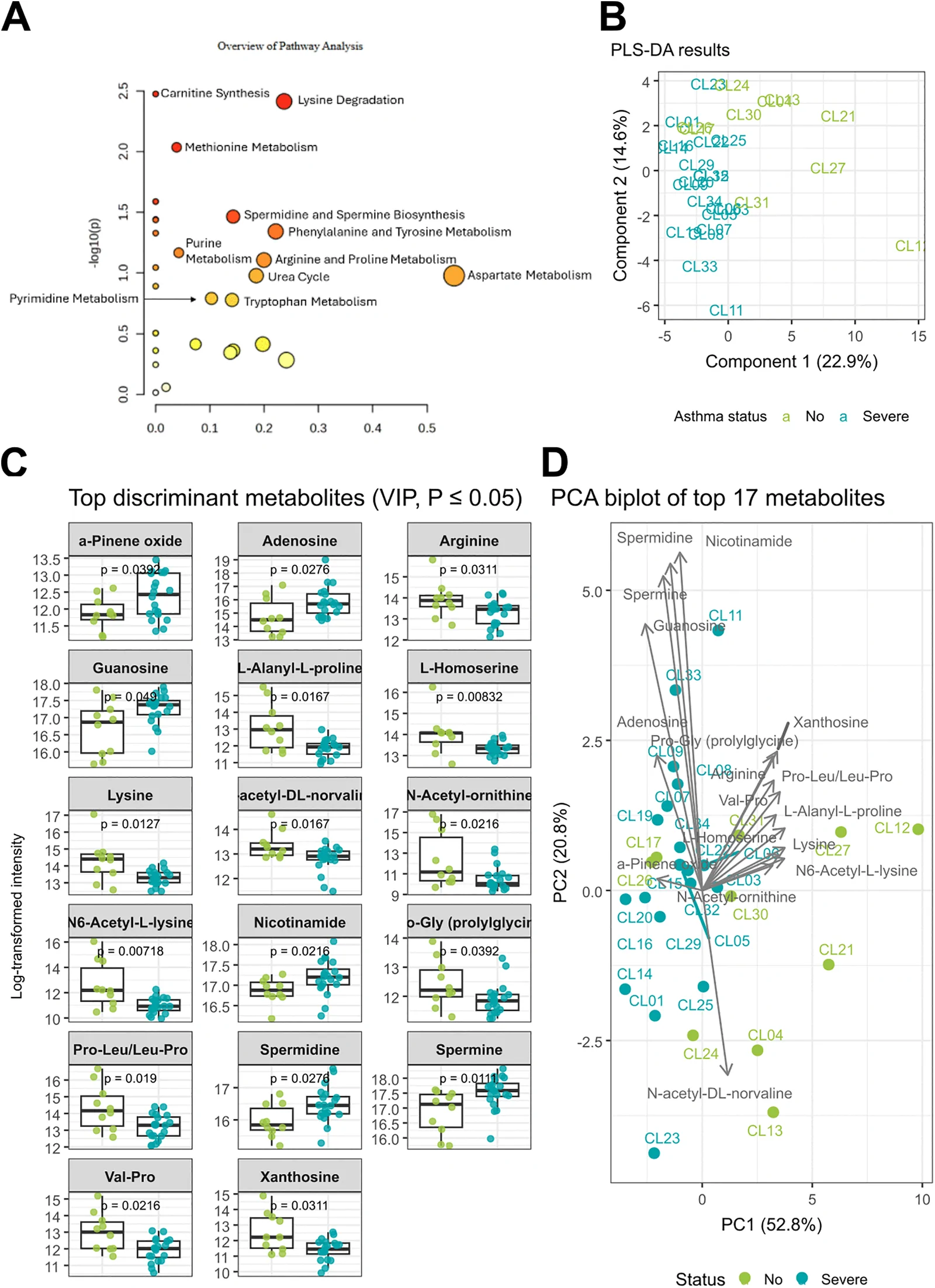
BAL metabolic signatures associated with severe asthma. A. Pathway analysis results from MetaboAnalyst. The scatter plot shows metabolic pathways with significance indicated by –log_10_(p-value) on the y-axis and pathway impact intensity on the x-axis. Each circle represents a pathway, with size proportional to pathway impact and colour intensity reflecting statistical significance. B. Partial Least Squares Discriminant Analysis (PLS-DA) score plot showing separation between SA and NA children based on metabolomic profiles. C. Barplots depicting the 17 metabolites that contribute most strongly to the model discriminating SA and NA individuals, ranked by combining Variable Importance in Projection (VIP) scores and Wilcoxon p-values (not adjusted). D. Principal Component Analysis (PCA) biplot of the top 17 discriminant metabolites, illustrating their contribution to variance and group separation.

We then compared the metabolomic profiles of BAL from SA and NA children, first via pathway analysis [42]. Several pathways were significantly enriched in SA compared with NA children (Fig 3A), including polyamine metabolism, carnitine synthesis, lysine degradation, and methionine metabolism (FDR-adjusted p < 0.1), suggesting potential metabolic dysregulation specific to SA. Although the impact scores of these pathways are relatively low, their statistical significance (FDR-adjusted p-values between 0.0398 and 0.0714) suggests biologically relevant alterations. The spermidine and spermine biosynthesis pathway showed moderate enrichment (FDR-adjusted p value = 0.113, 4/14 matched metabolites, impact score = 0.14). Pathways such as purine metabolism, urea cycle, and phenylalanine/tyrosine metabolism did not reach significance but showed trends that may reflect broader disruptions in nitrogen and amino acid metabolism, warranting further investigation in larger cohorts.

Finally, potential metabolites associated with severe asthma were examined using a supervised Partial Least Squares Discriminant Analysis (PLS-DA) model to differentiate SA and NA patients based on their metabolomic profiles. This method was selected for its effectiveness in handling high-dimensional datasets, which are typical in metabolomics [43,44]. The model demonstrated strong performance, with a sensitivity of 0.95 and a specificity of 0.70, yielding an overall classification accuracy of 0.867. Component 1 alone captured most of the discriminative signal (AUC = 0.86), while component 2 added limited value independently (AUC = 0.77). However, combining both components (via average score) substantially improved classification, reaching an AUC of 0.96 (S3 Fig). These results indicate that the PLS-DA model effectively discriminates between SA and NA groups. The model correctly identified 7 out of 10 NA individuals. The remaining 3 NA subjects, “CL17”, “CL26”, and “CL31”, were misclassified as SA (Fig 3B): two of the three misclassified individuals had chronic bronchopathy, and one had both tracheobronchomalacia and chronic bronchopathy. In contrast, 19 of 20 SA children were correctly classified. Subject “CL23” was misclassified as NA, despite presenting no clear clinical differences compared to other SA patients. This isolated misclassification further supports the robustness of the model while also highlighting the biological and clinical heterogeneity within asthma phenotypes. To identify the specific metabolic features driving group separation, we examined the Variable Importance in Projection (VIP) scores. The top 10 contributing metabolites were: N6-acetyl-lysine, alanyl-L-proline, N-acetyl-norvaline, lysine, Val-Pro, xanthosine, spermine, Pro-Leu/Leu-Pro, homoserine, and spermidine. Further characterisation of the metabolic differences between SA and NA, using univariate analysis with the Wilcoxon rank-sum test across all 88 annotated metabolites, revealed 17 nominally different metabolites (p < 0.05), although none remained significant after FDR correction (Fig 3C). Given the potential for type II error in the context of small sample sizes and exploratory metabolomic analyses, we opted for a combined approach that integrated both univariate and multivariate criteria. Specifically, we retained metabolites with an uncorrected Wilcoxon p-value < 0.05 and a VIP score > 1. This intersection highlighted 17 metabolites (S5 Table and Fig 3C), which we selected as candidates for further investigations. Among these, spermine and spermidine were significantly increased in severe asthma, while several dipeptides and amino acid derivatives were decreased, including alanyl-proline, homoserine, lysine, N-acetyl-norvaline, N6-acetyl-lysine, Pro-Leu/Leu-Pro, Val-Pro, and xanthosine. These findings suggest that polyamine metabolism and amino acid pathways may contribute to the pathophysiology of asthma. Unsupervised PCA based on the peak intensities of these 17 metabolites revealed a clear separation between the SA and NA groups along the first principal component (PC1), which accounted for 52.8% of the variance (Fig 3D). The same SA subject previously highlighted (CL23) appeared distant from the main SA cluster, positioned separately in the lower region of the PCA plot. This separation reflects a distinct metabolic profile characterized by lower levels of spermine and spermidine, similar to that observed in NA subjects. A few NA subjects (“CL17” and “CL26”) were positioned closer to the SA cluster, showing generally low metabolite intensities but relatively higher spermine and spermidine levels compared to other NA samples. Another NA sample (“CL31”) occupied an intermediate position, highlighting metabolic heterogeneity within the NA group. The PCA biplot demonstrates that these 17 metabolites capture the main variance driving group discrimination while revealing individual variability within each group.

### Microbiota-metabolites association analysis

**Fig 4:**
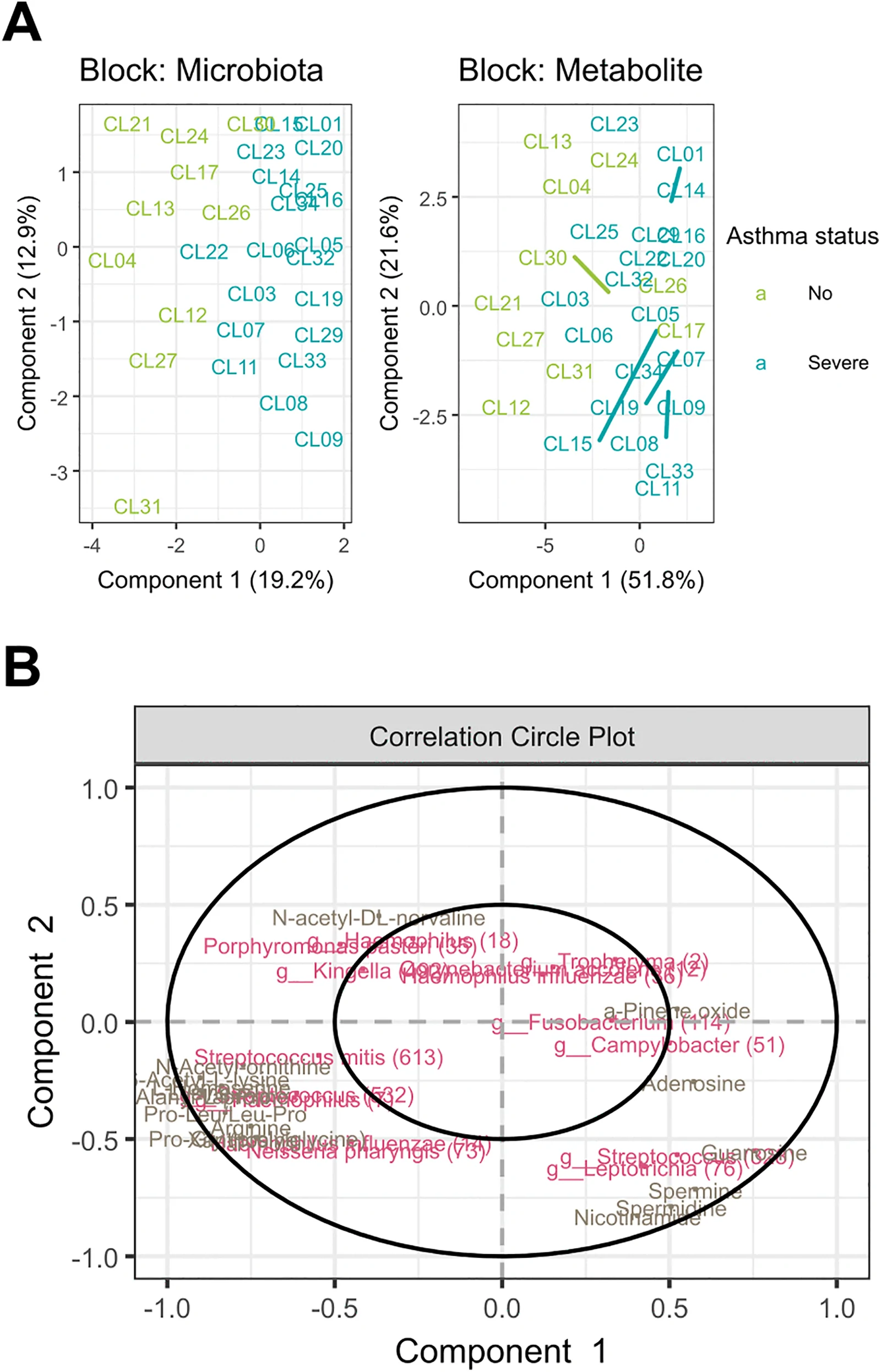
DIABLO results for microbiota–metabolite data integration. A. Projection of patients onto the first two latent components of the DIABLO model. BAL from SA (blue) and NA children (green) are clearly separated in the integrated multi-omics space. B. Correlation circle plot of selected variables. ASVs are shown in pink, and metabolites are shown in brown. Variables near the outer circle are highly correlated with the discriminant components, indicating their contribution to group separation. A coherent structure of microbial and metabolic features suggests inter-block correlations.

Then, a multiblock approach using the selected 17 metabolites and 15 ASVs, integrated in the DIABLO model, enabled clear separation of patients by their asthma status. In cross-validation (M-fold), the model demonstrated promising classification performance. Using Mahalanobis distance on the second latent component, the classification error was as low as 10.3% for the microbiome block and ranged from 12.7% to 16.3%, depending on the method applied, for the metabolite block. Evaluation, based on the mean of latent components, yielded strong predictive metrics: an overall accuracy of 90% (95% CI: 73.5–97.9%), a Cohen’s kappa coefficient of 0.77, a high specificity (95%), and a sensitivity of 80%. These results indicate that multi-omics integration effectively distinguished SA patients from NA individuals. Projection of samples onto the first two latent components revealed a clear separation into distinct groups (Fig 4A), reinforcing the relevance of the integrated analysis. The variable correlation circle (Fig 4B) showed a coherent structure across both data blocks, with distinct clusters of ASVs and metabolites that contributed to group discrimination. On the positive side of Component 1, several metabolites, including guanosine, spermine, spermidine, nicotinamide, α-pinene oxide, and adenosine, clustered together, indicating strong co-variation among these purine- and polyamine-related metabolites. Interestingly, *Streptococcus* (328), *Leptotrichia* (76), *Campylobacter* (51), *Fusobacterium* (114), and *Tropheryma* (2) were also located on this positive side, suggesting a positive association between these microbes and metabolites. In contrast, on the negative side of Component 1, a cluster of metabolites, including N6-acetyl-lysine, N-acetyl-ornithine, Pro-Gly, lysine, and arginine, co-located with several taxa from the *Haemophilus* (ASVs 7 and 14) and *Streptococcus* (ASVs 613 and 532) genera. Component 2 (y-axis) further separated variables within these clusters, with metabolites such as N-acetyl-norvaline positioned at the top, indicating their unique contribution to this axis. Taxa such as *P. pasteri* (35) and *Haemophilus* (18) also showed positive values on this component. However, Component 2 contributed less to differentiating the two patient groups than Component 1. These results suggest coordinated microbial–metabolite interactions potentially linked to bacterial community functional organization and SA status.

### Bacterial and metabolic characterisation of severe asthma sub-groups

#### Microbial and Metabolic Features Underlying Severe Asthma Sub-Phenotypes.

We previously showed in the same SA cohort that a specific set of immune components is associated with a clinical SA sub-phenotype characterized by frequent exacerbations (FE, defined as ≥ 3 severe exacerbations in the past year) [31], or by co-occuring atopic dermatitis [45]. This prompted us to investigate whether local bacterial taxa and/or metabolites may also serve as signatures of particular SA sub-phenotypes, specifically the co-occurrence of FE or of atopic dermatitis (AD).

**Fig 5:**
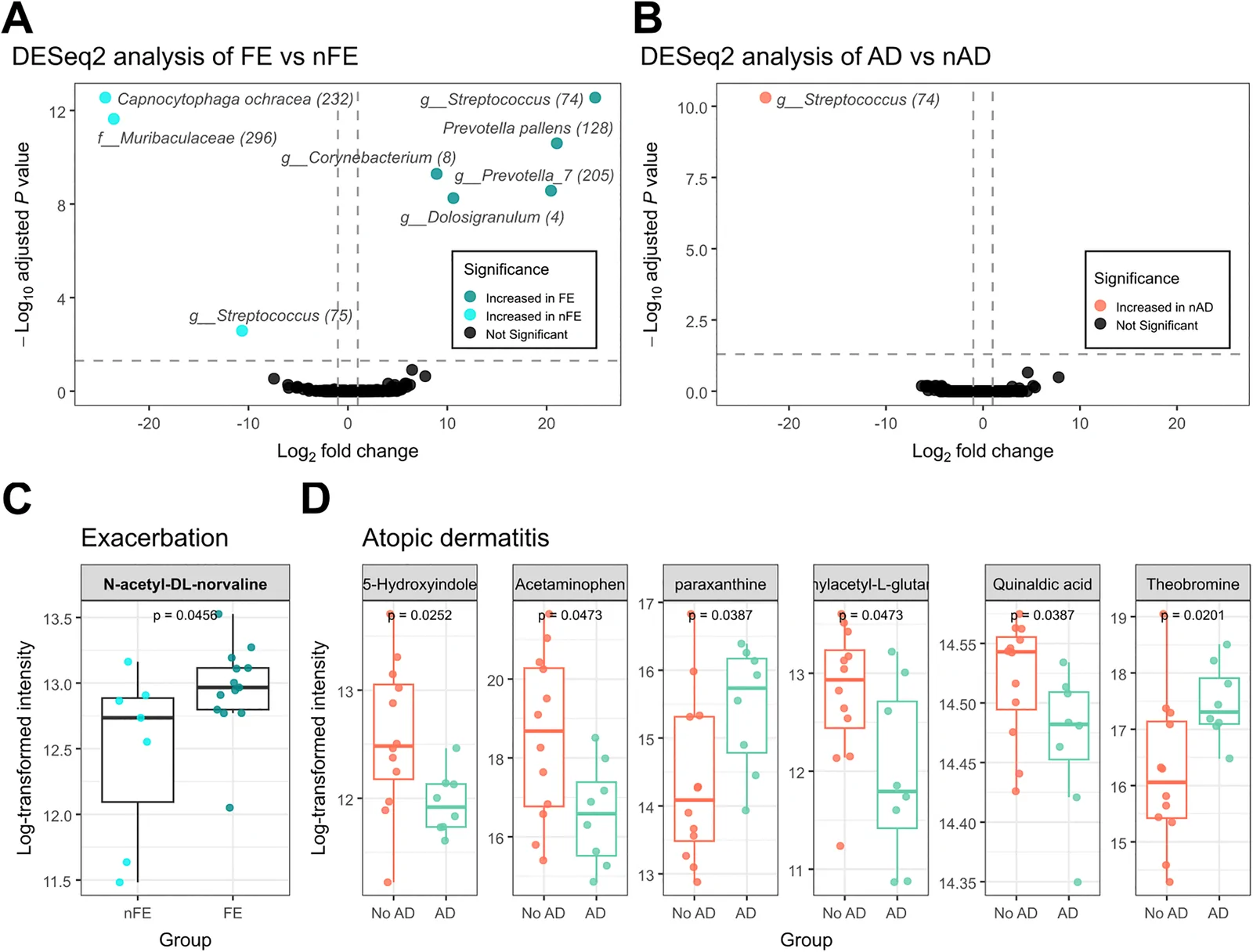
Distinct microbiota and metabolite signatures associated with severe asthma sub-phenotypes. A. Volcano plot showing differentially abundant bacterial taxa in children with SA who experienced frequent exacerbations (FE, ≥ 3 severe exacerbations in the past year, dark blue) compared with non-FE SA children (light blue), identified using DESeq2. B. Volcano plot showing differentially abundant bacterial taxa in SA children with or without AD (orange). C. Metabolite increased in SA-FE children, compared with SA-nFE, identified using a combined PLS-DA and Wilcoxon approach. D. Metabolites altered in SA children with (green) or without (orange) AD, identified using a combined PLS-DA and Wilcoxon approach.

Differential abundance analyses of bacterial taxa, in SA children only, revealed that SA children with FEs exhibited increased abundances of *Streptococcus* (74), *P. pallens* (128), *Prevotella 7* (205), *Dolosigranulum* (4), and *Corynebacterium* (8), and decreased relative abundances of *C. ochracea* (232), *Muribaculaceae* (296), and *Streptococcus* (75) (Fig 5A). In contrast, only *Streptococcus* (74) was associated with the absence of AD in SA children (Fig 5B, “nAD”). Interestingly, using the combined VIP and Wilcoxon approach described previously, only one metabolite - N-acetyl-norvaline - was elevated in SA-FE children (Fig 5C). Conversely, among all annotated metabolites, SA children with AD showed decreased abundances of 5-hydroxyindole, acetaminophen, phenylacetyl-glutamine, and quinaldic acid, compared with non-AD SA children, while paraxanthine and theobromine were increased (Fig 5D). Overall, these results reveal distinct microbiota and metabolite signatures that reflect the clinical heterogeneity of severe asthma.

### Unsupervised cluster analyses

**Fig 6:**
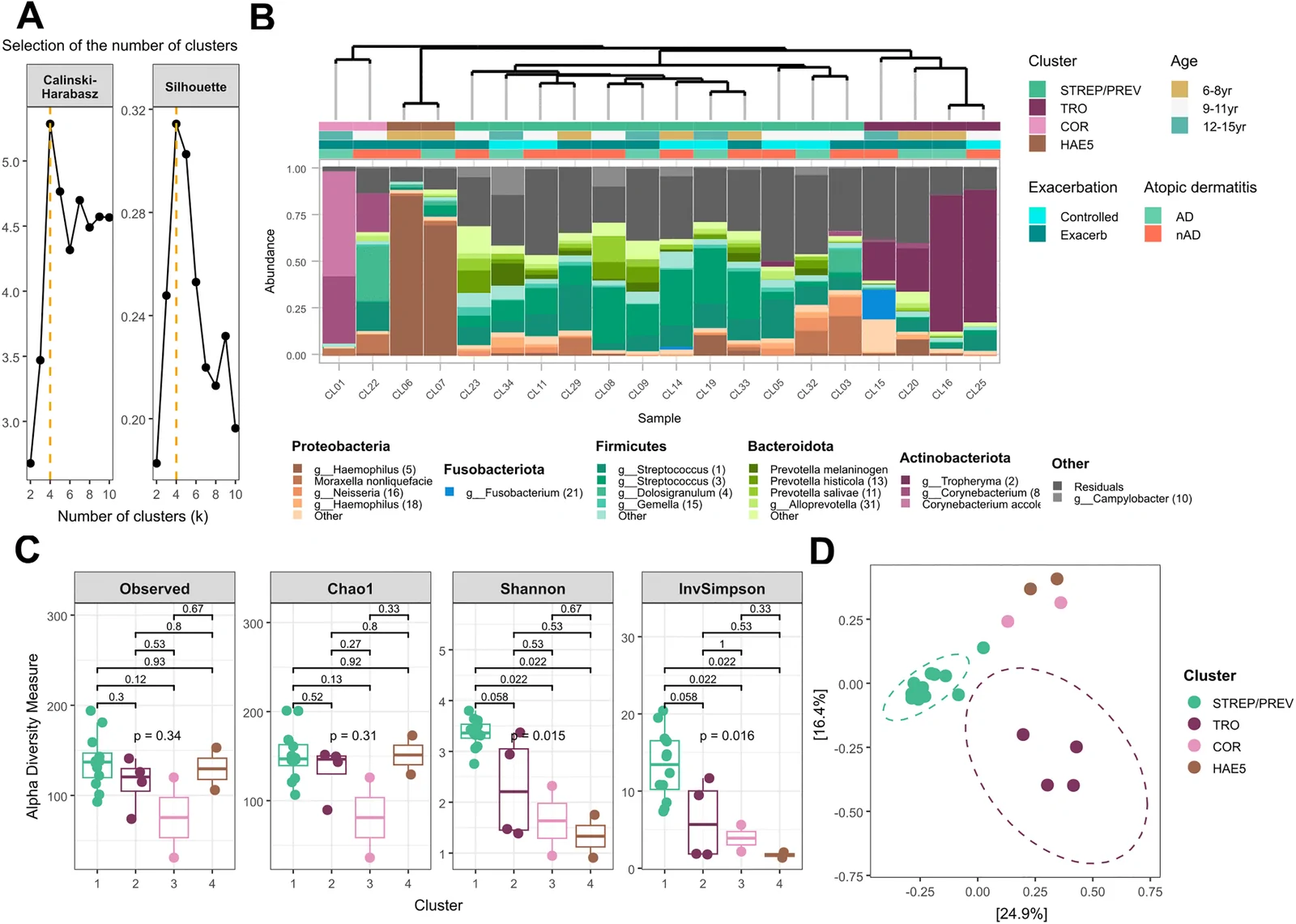
Unsupervised clustering analysis of BAL bacterial ASVs from children with SA. A. Calinski-Harabasz score and Silhouette coefficient plots used to determine the optimal number of SA sub-groups (clusters) based on bacterial ASVs detected in BAL. The orange dotted line indicates the optimal value beyond which the scores began to decrease. The optimal number of clusters was determined as k = 4. B. Hierarchical clustering. Clustering was performed using Bray-Curtis dissimilarity indices with average linkage. The top bars show cluster membership, age category, frequent exacerbation, and atopic dermatitis status for each individual. The compositional bars represent the relative abundances of the 25 most abundant ASVs across all 20 BAL samples. C. Alpha diversity measures (observed ASVs, Chao1 richness estimator, Shannon diversity index, and inverse Simpson index) for the four clusters. D. Principal coordinates analysis (PCoA) of Bray–Curtis dissimilarity, showing beta diversity patterns among the four clusters. Points represent individual samples, coloured by cluster membership. Ellipses represent 95% confidence intervals of the multivariate t-distribution for each group, illustrating the dispersion of samples around their group centroids in the ordination space.

Hierarchical clustering of Bray–Curtis dissimilarities indicated that the microbial communities grouped into k = 4 clusters. Actually, both the Calinski–Harabasz index and the average silhouette width reached their maxima at k = 4 (Fig 6A), supporting this solution as the most appropriate representation of the data. These clusters were dominated by a *Streptococcus/Prevotella* mix (STREP/PREV, n = 12), *Tropheryma* (TRO, n = 4), *Corynebacterium* (COR, n = 2) or *Haemophilus* (ASV5) (HAE5, n = 2) (Fig 6B). Interestingly, alpha diversity differed among microbial clusters identified by hierarchical clustering (Fig 6C). Although observed richness and Chao1 indices did not vary among clusters, Shannon diversity and inverse Simpson indices were significantly lower in clusters 3 (COR) and 4 (HAE5) than in Cluster 1 (STREP/PREV). Clusters with higher relative abundance of the STREP/PREV mix generally exhibited higher richness and diversity (although the differences were not significant for Observed Richness and the Chao1 index), suggesting a relationship between community composition and within-sample diversity. Cluster assignments overlaid on PCoA ordinations illustrated the distinct grouping of samples, particularly clusters 1 (STREP/PREV) and 2 (TRO) (Fig 6D), indicating that SA children segregated into four distinct subgroups, each dominated by an ASV, that may represent a particular endotype. However, given the small number of individuals in each cluster, particularly in the smaller groups, the statistical power to detect meaningful associations with clinical or phenotypical features was limited. Interestingly, repeating the clustering analysis on genus-level agglomerated data yielded the same number of clusters, each characterized by the same dominant taxa, indicating consistency across taxonomic resolutions. The overall cluster composition remained stable, except for one individual who was reclassified from the COR cluster to the STREP/PREV cluster. We also performed unsupervised clustering on the metabolite dataset. However, the Calinski–Harabasz index and average silhouette score produced inconsistent estimates of the optimal number of clusters, indicating that the metabolite data did not support a well-defined partitioning within the SA population in our cohort. Overall, the concordance between ASV- and genus-level clustering supports the stability of the microbial community structure observed in this SA cohort of children. In contrast, the less distinct clustering observed in the metabolite data suggests that larger cohorts will be required to resolve potential metabolic associations.

## Discussion

In this study, we provided an integrated characterization of the BAL microbiota and metabolites in a small but well-phenotyped cohort of children with severe asthma, revealing distinct microbial–metabolic signatures compared with non-asthmatic controls. Multi-omic integration of variables previously selected from each block further improved the prediction of SA vs non-SA status. Moreover, microbiota and metabolite signatures differed among SA children based on clinical sub-phenotypes including frequent exacerbations and co-occurrence of atopic dermatitis, respectively. Finally, distinct endotypes may be defined within SA children based on BAL microbiota composition.

We first observed an increase in the Chao1 alpha diversity metric in BALs from SA children compared to those from NA. While there appears to be a consensus that a decreased alpha-diversity of the gut microbiome is linked to a poorer health status, there is no clear evidence on how this generalizes to the microbiome of the lungs [46]. Indeed, our result is consistent with reports from other groups showing increased alpha diversity metrics in nasopharyngeal, induced sputum or bronchial brushing samples from asthmatic patients [39,47–50]. However, there is currently no consensus on whether alpha diversity metrics of the lower airway microbiota are increased, unchanged, or decreased in individuals with asthma compared to healthy controls [51]. These inconsistencies may reflect heterogeneity in patient age, disease severity, and sampling time (exacerbation period) or in analytical approaches.

Next, we identified a shift in bacterial composition in BALs from children with SA compared to controls. A key finding in our dataset was the increased relative abundance of the *Actinobacteriota* phylum, as well as the enrichment of taxa such as *Corynebacterium*, *Bifidobacterium*, and *Tropheryma*, all of which belong to the *Actinobacteriota* phylum, in SA compared to NA. Enrichment of this phylum in lower airways has already been reported by Huang *et al.* in adults with severe asthma [21]. Although in their cohort this was associated with the levels of FK506 Binding Protein 5 gene expression in bronchial brushings, a marker of steroid response, in our cohort, no clinical (nor demographic) features were associated with the observed variation in microbial community composition among samples. We also identified one ASV (ASV328, belonging to the genus *Streptococcus*) that was significantly increased in the airways of SA children across five distinct differential abundance analysis methods. This suggests that its enrichment is a consistent and reproducible feature of the SA airway microbiome in children, rather than an artifact of any single analytical approach. Consistent with previous reports in children and adults with asthma, severe asthma or recurrent wheezing, we also found an increased relative abundance of *Moraxella* in BAL from SA children [24,52,53]. However, contrary to previous reports [23,24], we did not observe a higher relative abundance of *Proteobacteria* in BAL samples from children with SA compared with NA. This finding could in part reflect the high prevalence of *Haemophilus* positivity in the NA group, with 40% of control children (4/10) showing positive bacteriology, which likely inflated *Proteobacteria* abundance in this group, whereas only 2 of 20 children with SA (10%) showed a positive bacteriology for *Haemophilus*.

Among the top 10 metabolites identified by orthogonal multi- and univariate methods, we found only two metabolites that were significantly increased in children with SA, namely spermine and spermidine. In line with our results, increased levels of these metabolites have been reported in the blood of asthmatic adults with active symptoms [54] and in the lung tissue of ovalbumin (OVA)-sensitized and challenged mice, which are widely used as an animal model of asthma [55,56]. Conversely, Wawrzyniak et al reported unchanged levels of spermine and decreased levels of spermidine in BALs from adults with asthma [57]. Furthermore, we have shown that arginine levels are reduced in SA, suggesting an increased metabolic flux from arginine to spermine and spermidine, thereby enhancing the polyamine pathway. Polyamines have been shown to regulate immune function. In particular, spermidine promotes the immunosuppressive phenotype of dendritic cells and the regulatory phenotype of CD4 + T cells, while reducing IL-17 production [58,59]. On the other hand, spermine was shown to promote eosinophil survival and activation in vitro [60].

Using the DIABLO framework, we found strong correlations between specific ASVs and metabolites. In particular, spermine and spermidine were associated with *Streptococcus* (ASV328) and *Leptotrichia*. Although polyamines appear to be important in lung physiology and pathophysiology, direct evidence that the lung microbiota produces polyamines that affect host lung cells is lacking. However, dysregulation of the polyamine pathway has already been linked to *Pseudomonas aeruginosa* infection, particularly to antibiotic resistance and bacterial virulence [61]. More importantly, *Streptococcus* (*pyogenes* and *pneumoniae*) may influence airway inflammation through polyamine production [62] or scavenging [63]. Because polyamines have been shown to enhance bacterial survival [63], reprogram macrophages toward an M2 phenotype [63], and promote a Th2 environment [64], these effects may lead to airway hyperreactivity, remodelling, and chronic inflammation. Our data thus provide new insights into the crosstalk between polyamine metabolism, microbiota and the immune response. This result clearly warrants further investigation of polyamine biosynthesis and uptake by lung-resident microbes and their effects on lung epithelial/immune cells.

Finally, our study has sought to provide a more detailled characterization of SA sub-phenotypes. We have shown that the microbiota dataset enabled a more precise profiling of BALs from SA children based on exacerbation frequency, whereas the metabolite dataset allowed differentiation of SA children based on co-occurring AD. SA children with frequent exacerbations showed increased relative abundances of *Prevotella*, *Corynebacterium* and *Dolosigranulum*. Interestingly, the latter two have been highlighted in asthma microbiota studies [64–66] as protective against symptom progression and exacerbation. These studies identified these bacteria in nasal or nasopharyngeal samples, with their abundances decreasing during exacerbation. Taken together with our results, this suggests that translocation of these taxa from the upper to the lower airways may rather reflect disease progression toward a more severe phenotype.

At the same time, our metabolomic analysis revealed that children with SA and co-occuring AD could be distinguished from children with SA without AD on the basis of specific metabolic profiles in the airways. Within the phenylalanine and tryptophan metabolic pathways, we observed decreased levels of phenylacetyl-glutamine, 5-hydroxyindole, and quinaldic acid in the SA-AD subgroup, suggesting a downregulation of indole- and phenylalanine-derived metabolites. Both 5-hydroxyindole and quinaldic acid belong to two major branches of the tryptophan metabolic network [67]. 5-hydroxyindole is a downstream product of the serotonin pathway, whereas quinaldic acid is linked to the kynurenine–quinolinic acid pathway. It has been shown that the activity of the indoleamine-2.3-dioxygenase (IDO)-1 and IDO-2 enzymes is impaired in patients with atopic conditions [67,68], resulting in reduced conversion of tryptophan into immunoregulatory metabolites such as kynurenine. This metabolite modulates multiple immune cell populations, promoting regulatory T cell differentiation while restraining Th17 and Th1 responses [67,69]. Although spermidine has been reported to induce IDO1 expression in dendritic cells [70], linking the polyamines and tryptophan metabolic pathways, in AD-SA patients, this regulatory axis appears uncoupled, as the kynurenine pathway seemed reduced despite elevated polyamines. Together, these findings highlight that children with SA and concomitant AD exhibit distinct airway metabolic signatures compared with SA children without AD, reflecting local – and maybe systemic – dysregulation of tryptophan-derived pathways. This suggests that SA subphenotypes are associated with local metabolic differences, which may contribute to the pathophysiology of atopic manifestations in severe asthma.

Such biomarkers may help to characterize further severe asthma endotypes. Our unsupervised clustering analysis of BAL from SA children revealed 4 microbiota patterns dominated by (i) *Streptococcus/Prevotella*, (ii)*Tropheryma*, (iii) *Corynebacterium*, and (iv) *Haemophilus*. Although 2 of 4 clusters were found in only 2 patients, *Streptococcus*-, *Haemophilus*-, and *Tropheryma*-dominated clusters have also been reported recently in sputum from SA adults using the relative-dominant species definition [71], although the proportions of SA patients in each cluster differed between our study and theirs. Another study by van Beveren *et al*. also identified *Corynebacterium*, *Haemophilus*, and *Streptococcus*-dominated clusters among others (*Moraxella*, *Dolosigranulum pigrum*, *Moraxella lincolnii*, and *Staphylococcus*) in nasopharyngeal samples from children, with the *Streptococcus*/mix cluster more prevalent in SA children than in controls [39]. Altogether, these results demonstrate that some SA endotypes may be defined through airway microbiota composition.

The main limitation of our study is the small cohort size, which precluded more comprehensive subgroup analyses integrating clinical, microbiological, immunological, and metabolic data. Additionally, due to ethical constraints, the control group comprised individuals with various underlying medical conditions rather than healthy controls. While differences in clinical characteristics between groups, particularly inhaled corticosteroid dosage and inflammatory parameters, may have influenced the observed microbiome and metabolome profiles, the heterogeneity of the control group did not preclude a precise and comprehensive characterization of the severe asthma group. In our study, no association was found between ICS dosage or eosinophil counts and bacterial taxa or metabolites. However, as these clinical parameters differed between SA and NA children (S1 Table), they may have contributed to the differences in microbiome and metabolites. As studies have previously shown that ICS and immune cells are associated with microbiome composition in airway disease [50,72–74], residual confounding cannot be fully excluded in this cross-sectional cohort, and future longitudinal or experimental studies are warranted to disentangle the independent effects of disease, treatment, and inflammation on the airway microbiome. Moreover, expanding metabolome coverage would further strengthen this study by enabling a more comprehensive characterization of SA-associated metabolic perturbations, for instance through the use of complementary LC-HRMS platforms (e.g., C18 and HILIC columns). Despite these limitations, the study has several notable strengths, including the use of age-matched pediatric controls, comprehensive phenotyping of all children and extensive metadata collection, such as information on domestic exposures, all of which enabled the identification of associations between microbial features and contextual factors. Importantly, analysing samples obtained directly from the affected tissue provides meaningful insights into airway-level mechanisms and highlights the value of tissue-specific profiling in deepening understanding of the disease and guiding therapeutic strategies. Upon further validation in larger cohorts, elucidating the role of these cluster-specific microbiome features in the progression of severe asthma or in the response to treatment may lead to the future development of biomarkers for early diagnosis and follow up. Future longitudinal and functional studies are warranted to clarify whether these dysbiotic features represent actionable biomarkers or potential therapeutic targets in severe pediatric asthma.

## Supporting information

S1 Table Demographic and clinical characteristics of the children with SA and the non-asthmatic disease controls (NA). (DOCX)

S2 Table Results of univariate PERMANOVA analyses evaluating the association between clinical, demographic, environmental, and treatment-related variables and the bronchoalveolar lavage (BAL) microbiota composition. Bray–Curtis dissimilarity was calculated from ASV-level relative abundance profiles, and each variable was tested separately using PERMANOVA (adonis2, vegan package) with 999 permutations. For each analysis, samples with missing values for the variable of interest were excluded. The table reports the proportion of variance explained (R²), nominal P value, and Benjamini–Hochberg false discovery rate (FDR)-adjusted P value (p-adj).
(DOCX)

S3 Table Significant associations identified by Pearson correlation analysis between clinical/demographic variables and metabolites. For each pathway–variable pair, the Pearson correlation coefficient and the unadjusted p-value are reported.
(DOCX)

S4 Table Significant associations identified by Pearson correlation analysis between clinical/demographic variables and metabolic pathways. For each metabolite–variable pair, the Pearson correlation coefficient and unadjusted p-value, and annotated KEGG pathways are reported.
(DOCX)

S5 Table Severe asthma (SA)–contributive metabolites identified by partial least squares discriminant analysis (PLS-DA). Metabolites are ranked by their mean variable importance in projection (VIP_mean; averaged across components 1 and 2). Corresponding p-values from univariate analyses (adjusted and unadjusted) are also reported.
(DOCX)

S1 Fig Principal coordinates analysis (PCoA) of Bray–Curtis dissimilarity on raw data (A), rarefied data (B), filtered data (C) and filtered rarefied data (D) showing beta diversity patterns between NA and SA samples. Points represent individual samples, coloured by asthma status. Ellipses indicate the dispersion of samples within each group, corresponding to one standard deviation from the group centroid in the multivariate space. The top 10 highest ranking ASVs are visualized as triangles.
(DOCX)

S2 Fig ASV- (left) and genera- (right) level differential abundance analyses using four statistical frameworks. (ANCOM, DESeq2, edgeR, and metagenomeSeq). Log fold changes (logFC) are plotted for each genus, with points representing individual ASVs or genera. Point color denotes phylum, and point size reflects statistical significance (−log10 FDR or -log10(adjusted p-value)). The vertical line indicates no differential abundance (logFC = 0), positive logFC values indicate enrichment in SA, whereas negative values indicate enrichment in NA.
(DOCX)

S3 Fig Receiver operating characteristic (ROC) curves for the first two components of PLS-DA. ROC curves were generated for component 1 (blue) and component 2 (orange), with the mean of the two components shown in green. The x-axis represents the false positive rate, and the y-axis represents the true positive rate. Diagonal dashed gray lines indicate the reference of no discrimination (AUC = 0.5). Area under the curve (AUC) values are reported in the legend for each component and the mean.
(DOCX)

## References

[1] Kuruvilla ME, Lee FE-H, Lee GB. Understanding asthma phenotypes, endotypes, and mechanisms of disease. Clinic Rev Allerg Immunol. 2019;56:219–33. doi: 10.1007/s12016-018-8712-1 PMC6411459 30206782

[2] Haktanir Abul M, Phipatanakul W. Severe asthma in children: Evaluation and management. Allergol Int. 2019;68(2):150–7. doi: 10.1016/j.alit.2018.11.007 30648539

[3] Adel-Patient K, Grauso M, Abou-Taam R, Guillon B, Dietrich C, Machavoine F, et al. A Comprehensive Analysis of Immune Constituents in Blood and Bronchoalveolar Lavage Allows Identification of an Immune Signature of Severe Asthma in Children. Front Immunol. 2021;12:700521. doi: 10.3389/fimmu.2021.700521 34349761 PMC8327906

[4] Ekşi M, Bulut H, Akalin E, Ustabaş Kahraman F, Yazan H, Yazici M, et al. Evaluation of the association of mucosa-associated invariant T (MAIT) cells with childhood asthma. Turk J Med Sci. 2024;54(6):1265–70. doi: 10.55730/1300-0144.5908 39734354 PMC11673616

[5] Wisniewski JA, Muehling LM, Eccles JD, Capaldo BJ, Agrawal R, Shirley D-A, et al. TH1 signatures are present in the lower airways of children with severe asthma, regardless of allergic status. J Allergy Clin Immunol. 2018;141(6):2048-2060.e13. doi: 10.1016/j.jaci.2017.08.020 28939412 PMC5860937

[6] Chung KF, Wenzel SE, Brozek JL, Bush A, Castro M, Sterk PJ, et al. International ERS/ATS guidelines on definition, evaluation and treatment of severe asthma. Eur Respir J. 2014;43(2):343–73. doi: 10.1183/09031936.00202013 24337046

[7] Manti S, Magri P, De Silvestri A, De Filippo M, Votto M, Marseglia GL, et al. Epidemiology of severe asthma in children: a systematic review and meta-analysis. Eur Respir Rev. 2024;33(174):240095. doi: 10.1183/16000617.0095-2024 39384302 PMC11462310

[8] Nordlund B, Melén E, Schultz ES, Grönlund H, Hedlin G, Kull I. Prevalence of severe childhood asthma according to the WHO. Respir Med. 2014;108(8):1234–7. doi: 10.1016/j.rmed.2014.05.015 24939389

[9] Asher MI, Rutter CE, Bissell K, Chiang C-Y, El Sony A, Ellwood E, et al. Worldwide trends in the burden of asthma symptoms in school-aged children: Global Asthma Network Phase I cross-sectional study. Lancet. 2021;398(10311):1569–80. doi: 10.1016/S0140-6736(21)01450-1 34755626 PMC8573635

[10] Castro-Rodriguez JA, Soto-Martínez ME, Rodriguez-Martinez CE, Mocelin HT, Fischer GB, Mallol J. Severe childhood asthma in low and middle-income countries. Paediatr Respir Rev. 2026;57:17–26. doi: 10.1016/j.prrv.2025.06.002 40731240

[11] Lezmi G, de Blic J. Assessment of airway inflammation and remodeling in children with severe asthma: The next challenge. Pediatr Pulmonol. 2018;53(9):1171–3. doi: 10.1002/ppul.24051 29766674

[12] Invernizzi R, Lloyd CM, Molyneaux PL. Respiratory microbiome and epithelial interactions shape immunity in the lungs. Immunology. 2020;160(2):171–82. doi: 10.1111/imm.13195 32196653 PMC7218407

[13] Hernandez-Leyva AJ, Rosen AL, Tomera CP, Lin EE, Akaho EH, Blatz AM, et al. Upper and lower airway microbiota across infancy and childhood. Pediatr Res. 2025;98(4):1449–59. doi: 10.1038/s41390-025-03942-0 40075175 PMC12549332

[14] Barcik W, Boutin RCT, Sokolowska M, Finlay BB. The role of lung and gut microbiota in the pathology of asthma. Immunity. 2020;52(2):241–55. doi: 10.1016/j.immuni.2020.01.007 32075727 PMC7128389

[15] Mathieu E, Marquant Q, Descamps D, Riffault S, Saint-Criq V, Thomas M. Le poumon est sensible aux effets locaux et à distance des microbiotes. Nutrition Clinique et Métabolisme. 2021;35(4):242–52. doi: 10.1016/j.nupar.2021.04.002

[16] Mathieu E, Escribano-Vazquez U, Descamps D, Cherbuy C, Langella P, Riffault S, et al. Paradigms of lung microbiota functions in health and disease, particularly, in asthma. Front Physiol. 2018;9: 1168. doi: 10.3389/fphys.2018.01168 PMC6110890 30246806

[17] Teo SM, Mok D, Pham K, Kusel M, Serralha M, Troy N, et al. The infant nasopharyngeal microbiome impacts severity of lower respiratory infection and risk of asthma development. Cell Host Microbe. 2015;17(5):704–15. doi: 10.1016/j.chom.2015.03.008 25865368 PMC4433433

[18] Teo SM, Tang HHF, Mok D, Judd LM, Watts SC, Pham K, et al. Airway microbiota dynamics uncover a critical window for interplay of pathogenic bacteria and allergy in childhood respiratory disease. Cell Host Microbe. 2018;24(3):341-352.e5. doi: 10.1016/j.chom.2018.08.005 30212648 PMC6291254

[19] Bosch AATM, de Steenhuijsen Piters WAA, van Houten MA, Chu MLJN, Biesbroek G, Kool J, et al. Maturation of the Infant Respiratory Microbiota, Environmental Drivers, and Health Consequences. A Prospective Cohort Study. Am J Respir Crit Care Med. 2017;196(12):1582–90. doi: 10.1164/rccm.201703-0554OC 28665684

[20] de Steenhuijsen Piters WAA, Sanders EAM, Bogaert D. The role of the local microbial ecosystem in respiratory health and disease. Philos Trans R Soc Lond B Biol Sci. 2015;370(1675):20140294. doi: 10.1098/rstb.2014.0294 26150660 PMC4528492

[21] Huang YJ, Nariya S, Harris JM, Lynch SV, Choy DF, Arron JR, et al. The airway microbiome in patients with severe asthma: Associations with disease features and severity. J Allergy Clin Immunol. 2015;136(4):874–84. doi: 10.1016/j.jaci.2015.05.044 26220531 PMC4600429

[22] Simpson JL, Daly J, Baines KJ, Yang IA, Upham JW, Reynolds PN, et al. Airway dysbiosis: Haemophilus influenzae and Tropheryma in poorly controlled asthma. Eur Respir J. 2016;47(3):792–800. doi: 10.1183/13993003.00405-2015 26647445

[23] Hilty M, Burke C, Pedro H, Cardenas P, Bush A, Bossley C, et al. Disordered microbial communities in asthmatic airways. PLoS One. 2010;5(1):e8578. doi: 10.1371/journal.pone.0008578 20052417 PMC2798952

[24] Green BJ, Wiriyachaiporn S, Grainge C, Rogers GB, Kehagia V, Lau L, et al. Potentially pathogenic airway bacteria and neutrophilic inflammation in treatment resistant severe asthma. PLoS One. 2014;9(6):e100645. doi: 10.1371/journal.pone.0100645 24955983 PMC4067344

[25] Chun Y, Do A, Grishina G, Grishin A, Fang G, Rose S, et al. Integrative study of the upper and lower airway microbiome and transcriptome in asthma. JCI Insight. 2020;5(5):e133707. doi: 10.1172/jci.insight.133707 32161195 PMC7141394

[26] Turi KN, Romick-Rosendale L, Ryckman KK, Hartert TV. A review of metabolomics approaches and their application in identifying causal pathways of childhood asthma. J Allergy Clin Immunol. 2018;141(4):1191–201. doi: 10.1016/j.jaci.2017.04.021 28479327 PMC5671382

[27] Papamichael MM, Katsardis C, Sarandi E, Georgaki S, Frima E-S, Varvarigou A. Application of Metabolomics in Pediatric Asthma: Prediction, Diagnosis and Personalized Treatment. Metabolites. 2021;11:251. doi: 10.3390/metabo11040251 PMC8072856 33919626

[28] Liang L, Hu M, Chen Y, Liu L, Wu L, Hang C, et al. Metabolomics of bronchoalveolar lavage in children with persistent wheezing. Respir Res. 2022;23(1):161. doi: 10.1186/s12931-022-02087-6 35718784 PMC9208141

[29] Rzetecka N, Matysiak J, Matysiak J, Sobkowiak P, Wojsyk-Banaszak I, Bręborowicz A, et al. Metabolomics in Childhood Asthma - a Promising Tool to Meet Various Clinical Needs. Curr Allergy Asthma Rep. 2025;25(1):24. doi: 10.1007/s11882-025-01198-6 40341431 PMC12062110

[30] Macowan M, Pattaroni C, Bonner K, Chatzis R, Daunt C, Gore M, et al. Deep multiomic profiling reveals molecular signatures that underpin preschool wheeze and asthma. J Allergy Clin Immunol. 2025;155(1):94–106. doi: 10.1016/j.jaci.2024.08.017 39214237

[31] Adel-Patient K, Grauso M, Abou-Taam R, Guillon B, Dietrich C, Machavoine F, et al. Immune signatures distinguish frequent from non-frequent exacerbators among children with severe asthma. Allergy. 2021;76(7):2261–4. doi: 10.1111/all.14759 33544926

[32] Singh A, Shannon CP, Gautier B, Rohart F, Vacher M, Tebbutt SJ, et al. DIABLO: an integrative approach for identifying key molecular drivers from multi-omics assays. Bioinformatics. 2019;35(17):3055–62. doi: 10.1093/bioinformatics/bty1054 30657866 PMC6735831

[33] Lezmi G, Abou-Taam R, Garcelon N, Dietrich C, Machavoine F, Delacourt C, et al. Evidence for a MAIT-17-high phenotype in children with severe asthma. J Allergy Clin Immunol. 2019;144(6):1714-1716.e6. doi: 10.1016/j.jaci.2019.08.003 31425779

[34] Briard M, Guillon B, Venot E, Grauso M, Bruneau A, Rossignol M-N, et al. Datasets of 16S rRNA gene amplicon sequences, metabolites, and soluble immune components in bronchoalveolar lavage samples from severe asthmatic and age-matched control children. Data Brief. 2025;64:112359. doi: 10.1016/j.dib.2025.112359 41503622 PMC12769396

[35] Adel-Patient K. Dataset of untargeted metabolomics analysis of bronchoalveolar lavage samples from severe asthmatic and age-matched control children. Recherche Data Gouv. 2025. doi: 10.57745/1L8VRI

[36] Saint-Criq V, Briard M. Dataset of 16S rRNA gene amplicon sequences in bronchoalveolar lavage samples from severe asthmatic and age-matched control children. Recherche Data Gouv. 2025. doi: 10.57745/LL3TFW PMC12769396 41503622

[37] Suzuki MT, Taylor LT, DeLong EF. Quantitative analysis of small-subunit rRNA genes in mixed microbial populations via 5’-nuclease assays. Appl Environ Microbiol. 2000;66(11):4605–14. doi: 10.1128/AEM.66.11.4605-4614.2000 11055900 PMC92356

[38] Mayeur C, Gratadoux J-J, Bridonneau C, Chegdani F, Larroque B, Kapel N, et al. Faecal D/L lactate ratio is a metabolic signature of microbiota imbalance in patients with short bowel syndrome. PLoS One. 2013;8(1):e54335. doi: 10.1371/journal.pone.0054335 23372709 PMC3553129

[39] van Beveren GJ, de Steenhuijsen Piters WAA, Boeschoten SA, Louman S, Chu ML, Arp K, et al. Nasopharyngeal microbiota in children is associated with severe asthma exacerbations. J Allergy Clin Immunol. 2024;153(6):1574-1585.e14. doi: 10.1016/j.jaci.2024.02.020 38467291

[40] Pang Z, Lu Y, Zhou G, Hui F, Xu L, Viau C, et al. MetaboAnalyst 6.0: towards a unified platform for metabolomics data processing, analysis and interpretation. Nucleic Acids Res. 2024;52(W1):W398–406. doi: 10.1093/nar/gkae253 38587201 PMC11223798

[41] Kloepfer KM, Kennedy JL. Childhood respiratory viral infections and the microbiome. J Allergy Clin Immunol. 2023;152(4):827–34. doi: 10.1016/j.jaci.2023.08.008 37607643 PMC10592030

[42] Lu Y, Pang Z, Xia J. Comprehensive investigation of pathway enrichment methods for functional interpretation of LC-MS global metabolomics data. Brief Bioinform. 2023;24(1):bbac553. doi: 10.1093/bib/bbac553 36572652 PMC9851290

[43] Correnti S, Preianò M, Gamboni F, Stephenson D, Pelaia C, Pelaia G, et al. An integrated metabo-lipidomics profile of induced sputum for the identification of novel biomarkers in the differential diagnosis of asthma and COPD. J Transl Med. 2024;22(1):301. doi: 10.1186/s12967-024-05100-2 38521955 PMC10960495

[44] Kelly RS, McGeachie MJ, Lee-Sarwar KA, Kachroo P, Chu SH, Virkud YV, et al. Partial Least Squares Discriminant Analysis and Bayesian Networks for Metabolomic Prediction of Childhood Asthma. Metabolites. 2018;8(4):68. doi: 10.3390/metabo8040068 30360514 PMC6316795

[45] Lezmi G, Poirault C, Grauso M, Dietrich C, Adel-Patient K, Leite-de-Moraes M. Identification of the major immune differences in severe asthmatic children according to their atopic dermatitis status. Cell Immunol. 2024;397–398:104815. doi: 10.1016/j.cellimm.2024.104815 38428350

[46] Avalos-Fernandez M, Alin T, Métayer C, Thiébaut R, Enaud R, Delhaes L. The respiratory microbiota alpha-diversity in chronic lung diseases: first systematic review and meta-analysis. Respir Res. 2022;23(1):214. doi: 10.1186/s12931-022-02132-4 35999634 PMC9396807

[47] Liu H-Y, Li C-X, Liang Z-Y, Zhang S-Y, Yang W-Y, Ye Y-M, et al. The Interactions of Airway Bacterial and Fungal Communities in Clinically Stable Asthma. Front Microbiol. 2020;11:1647. doi: 10.3389/fmicb.2020.01647 32849339 PMC7396634

[48] Huang YJ, Nelson CE, Brodie EL, Desantis TZ, Baek MS, Liu J, et al. Airway microbiota and bronchial hyperresponsiveness in patients with suboptimally controlled asthma. J Allergy Clin Immunol. 2011;127(2):372-381.e1-3. doi: 10.1016/j.jaci.2010.10.048 21194740 PMC3037020

[49] Marri PR, Stern DA, Wright AL, Billheimer D, Martinez FD. Asthma-associated differences in microbial composition of induced sputum. J Allergy Clin Immunol. 2013;131(2):346-52.e1-3. doi: 10.1016/j.jaci.2012.11.013 23265859 PMC4403876

[50] Denner DR, Sangwan N, Becker JB, Hogarth DK, Oldham J, Castillo J, et al. Corticosteroid therapy and airflow obstruction influence the bronchial microbiome, which is distinct from that of bronchoalveolar lavage in asthmatic airways. J Allergy Clin Immunol. 2016;137(5):1398-1405.e3. doi: 10.1016/j.jaci.2015.10.017 26627545 PMC4860110

[51] Versi A, Ivan FX, Abdel-Aziz MI, Bates S, Riley J, Baribaud F, et al. Haemophilus influenzae and Moraxella catarrhalis in sputum of severe asthma with inflammasome and neutrophil activation. Allergy. 2023;78(11):2906–20. doi: 10.1111/all.15776 37287344

[52] Al Bataineh MT, Hamoudi RA, Dash NR, Ramakrishnan RK, Almasalmeh MA, Sharif HA, et al. Altered respiratory microbiota composition and functionality associated with asthma early in life. BMC Infect Dis. 2020;20(1):697. doi: 10.1186/s12879-020-05427-3 32962658 PMC7510324

[53] Zhang L, Ai T, Xie C, Xia W, Zhang Y, Liao H, et al. Lower airway microbiome of children with recurrent wheezing: a clinical cohort study. Transl Pediatr. 2022;11(5):696–705. doi: 10.21037/tp-22-165 35685081 PMC9173878

[54] Kurosawa M, Shimizu Y, Tsukagoshi H, Ueki M. Elevated levels of peripheral-blood, naturally occurring aliphatic polyamines in bronchial asthmatic patients with active symptoms. Allergy. 1992;47(6):638–43. doi: 10.1111/j.1398-9995.1992.tb02388.x 1285570

[55] North ML, Grasemann H, Khanna N, Inman MD, Gauvreau GM, Scott JA. Increased ornithine-derived polyamines cause airway hyperresponsiveness in a mouse model of asthma. Am J Respir Cell Mol Biol. 2013;48(6):694–702. doi: 10.1165/rcmb.2012-0323OC 23470627

[56] Jain V, Raina S, Gheware AP, Singh R, Rehman R, Negi V, et al. Reduction in polyamine catabolism leads to spermine-mediated airway epithelial injury and induces asthma features. Allergy. 2018;73(10):2033–45. doi: 10.1111/all.13472 29729200 PMC6461714

[57] Wawrzyniak M, Groeger D, Frei R, Ferstl R, Wawrzyniak P, Krawczyk K, et al. Spermidine and spermine exert protective effects within the lung. Pharmacol Res Perspect. 2021;9(4):e00837. doi: 10.1002/prp2.837 34289267 PMC8294051

[58] Li G, Ding H, Yu X, Meng Y, Li J, Guo Q, et al. Spermidine Suppresses Inflammatory DC function by activating the FOXO3 pathway and counteracts autoimmunity. iScience. 2020;23(1):100807. doi: 10.1016/j.isci.2019.100807 31962236 PMC6971394

[59] Carriche GM, Almeida L, Stüve P, Velasquez L, Dhillon-LaBrooy A, Roy U, et al. Regulating T-cell differentiation through the polyamine spermidine. J Allergy Clin Immunol. 2021;147(1):335-348.e11. doi: 10.1016/j.jaci.2020.04.037 32407834

[60] Ilmarinen P, Moilanen E, Erjefält JS, Kankaanranta H. The polyamine spermine promotes survival and activation of human eosinophils. J Allergy Clin Immunol. 2015;136(2):482-4.e11. doi: 10.1016/j.jaci.2014.12.1922 25649081

[61] Moyne O, Castelli F, Bicout DJ, Boccard J, Camara B, Cournoyer B, et al. Metabotypes of Pseudomonas aeruginosa Correlate with Antibiotic Resistance, Virulence and Clinical Outcome in Cystic Fibrosis Chronic Infections. Metabolites. 2021;11(2):63. doi: 10.3390/metabo11020063 33494144 PMC7909822

[62] Nanduri B, Swiatlo E. The expansive effects of polyamines on the metabolism and virulence of Streptococcus pneumoniae. Pneumonia (Nathan). 2021;13(1):4. doi: 10.1186/s41479-021-00082-x 33762024 PMC7990898

[63] Banerji R, Iyer P, Saroj SD. Spermidine enhances the survival of Streptococcus pyogenes M3 under oxidative stress. Mol Oral Microbiol. 2022;37(2):53–62. doi: 10.1111/omi.12360 34994090

[64] Zhou Y, Jackson D, Bacharier LB, Mauger D, Boushey H, Castro M, et al. The upper-airway microbiota and loss of asthma control among asthmatic children. Nat Commun. 2019;10(1):5714. doi: 10.1038/s41467-019-13698-x 31844063 PMC6915697

[65] Hou J, Song Y, Leung ASY, Tang MF, Shi M, Wang EY, et al. Temporal dynamics of the nasopharyngeal microbiome and its relationship with childhood asthma exacerbation. Microbiol Spectr. 2022;10(3):e0012922. doi: 10.1128/spectrum.00129-22 35546575 PMC9241764

[66] McCauley K, Durack J, Valladares R, Fadrosh DW, Lin DL, Calatroni A, et al. Distinct nasal airway bacterial microbiotas differentially relate to exacerbation in pediatric patients with asthma. J Allergy Clin Immunol. 2019;144(5):1187–97. doi: 10.1016/j.jaci.2019.05.035 31201890 PMC6842413

[67] Gabela AM, Mthembu N, Hadebe S. Tryptophan metabolism in health and disease- implications for non-communicable diseases. Immunol Lett. 2026;277:107093. doi: 10.1016/j.imlet.2025.107093 41015394

[68] Huang Y, Chen G, Liu X, Shao Y, Gao P, Xin C, et al. Serum metabolomics study and eicosanoid analysis of childhood atopic dermatitis based on liquid chromatography–mass spectrometry. J Proteome Res. 2014;13:5715–23. doi: 10.1021/pr5007069 25316199

[69] De Araújo EF, Feriotti C, Galdino NADL, Preite NW, Calich VLG, Loures FV. The IDO–AhR axis controls Th17/Treg Immunity in a Pulmonary Model of Fungal Infection. Front Immunol. 2017;8:880. doi: 10.3389/fimmu.2017.00880 PMC5523665 28791025

[70] Mondanelli G, Bianchi R, Pallotta MT, Orabona C, Albini E, Iacono A, et al. A Relay Pathway between Arginine and Tryptophan Metabolism Confers Immunosuppressive Properties on Dendritic Cells. Immunity. 2017;46(2):233–44. doi: 10.1016/j.immuni.2017.01.005 28214225 PMC5337620

[71] Versi A, Azim A, Ivan FX, Abdel-Aziz MI, Bates S, Riley J, et al. A severe asthma phenotype of excessive airway Haemophilus influenzae relative abundance associated with sputum neutrophilia. Clin Transl Med. 2024;14(9):e70007. doi: 10.1002/ctm2.70007 39187935 PMC11347389

[72] Dicker AJ, Huang JTJ, Lonergan M, Keir HR, Fong CJ, Tan B, et al. The sputum microbiome, airway inflammation, and mortality in chronic obstructive pulmonary disease. J Allergy Clin Immunol. 2021;147(1):158–67. doi: 10.1016/j.jaci.2020.02.040 32353489

[73] Singanayagam A, Glanville N, Cuthbertson L, Bartlett NW, Finney LJ, Turek E, et al. Inhaled corticosteroid suppression of cathelicidin drives dysbiosis and bacterial infection in chronic obstructive pulmonary disease. Sci Transl Med. 2019;11(507):eaav3879. doi: 10.1126/scitranslmed.aav3879 31462509 PMC7237237

[74] Richardson H, Alferes De Lima Headley D, Clarke C, Veluchamy A, Rauchhaus P, Pollock J, et al. The effect of different inhaled corticosteroid and long-acting bronchodilator combinations on the airway microbiome in patients with severe COPD: a randomised trial (MUSIC). Eur Respir J. 2025;66(4):2500287. doi: 10.1183/13993003.00287-2025 40841147

[75] MIGALE. INRAE. 2018. doi: 10.15454/1.5572390655343293E12

