# Supplementary material for: A microbiome–metabolome signature associated with pediatric severe asthma
Source: PLoS One. 2026 Sep 18;21(9):e0358560. doi: 10.1371/journal.pone.0358560 (PMC13588362; doi:10.1371/journal.pone.0358560)
Supplement: Supplementary file 1 [file pone.0358560.s001.docx]

**S1 Table**

| **Study participants, n** |  | **Severe Asthma (SA)** |  | **Non-Asthmatic (NA)** | **p-value NA vs SA** |
| --- | --- | --- | --- | --- | --- |
|  |  | **20** |  | **10** |  |
| Age (years) |  | 10.5 (7.7-12.4) |  | 10.7 (7.7-12) | 0.82 |
| Gender (female:male) |  | 07:15 |  | 04:06 | 1* |
| BMI (kg/m²) |  | 17.6 (15.5-23.1) |  | 17.7 (16.2-19.8) | 0.98 |
| Atopy history, n |  | 17/20 |  | 6/10 | 0.18* |
| Post-BD FEV1 (%) |  | 109 (97-116) |  | 92 (86-102; n=9) | 0.14 |
| Post-BD FEV1/FVC (%) |  | 87.5 (81.2-90.5) |  | 76 (73-86; n=9) | ***0.057*** |
| ACT score |  | 17.5 (13-21.2) |  | 19 (15-21; n=5) | 0.78 |
| Number of bursts |  | 5 (2-9) |  | 1 (0-2; n=5) | 0.15 |
| ICS (μg/day eq. fluticasone) |  | 500 (500-562.5) |  | 300 (250-500) | **0.007** |
| Circulating Total IgE (UI/ml) |  | 218 (136.8-671) |  | 43.57 (36.9-281.9) | 0.15 |
| Blood leucocytes (x103/μl) |  | 6.7 (5.1-7.4) |  | 7.05 (6.3-10.2) | 0.24 |
| Blood eosinophils (x103/μl) |  | 0.5 (0.3-0.9) |  | 0.2 (0.17-0.32) | **0.008** |
| Blood neutrophils (x103/μl) |  | 2.7 (1.9-3.4) |  | 3.8 (2-6.8) | 0.21 |
| Blood lymphocytes (x103/μl) |  | 2.5 (1.9-2.9) |  | 2.65 (2.5-2.8) | 0.65 |
| BAL eosinophils % |  | 0 (0-0) |  | 0 (0-0) | 0.35 |
| BAL neutrophils % |  | 2 (1-4) |  | 38 (15-84) | **0.001** |
| BAL lymphocytes % |  | 6 (5-8) |  | 4 (3-5) | ***0.059*** |
| Positive BAL bacteriology, n |  | 5/20 |  | 5/10 | 0.23* |
| Positive BAL virology, n |  | 4/20 |  | 1/10 | 0.64* |
|  |  |  |  |  |  |
| *BMI, body mass index; BD, bronchodilator; FEV1, forced expiratory volume (1 s); FVC, forced vital capacity; ACT, asthma control test. Post-BD FEV, ACT, and the number of bursts were not available for all controls (number indicated within brackets). Median values (and inter-quartile ranges) are provided for quantitative values. Statistical analysis: groups were compared using the MW test (1,000 Monte Carlo simulations) or Fisher’s exact test (indicated by an *). Bold values are statistically significant (P < 0.05). Trends are indicated by italics.* | | | | | |
