# Supplementary material for: A microbiome–metabolome signature associated with pediatric severe asthma
Source: PLoS One. 2026 Sep 18;21(9):e0358560. doi: 10.1371/journal.pone.0358560 (PMC13588362; doi:10.1371/journal.pone.0358560)
Supplement: Supplementary file 2 [file pone.0358560.s002.docx]

**S2 Table**

| **Variable** | **R2** | **p-value** | **p-adj** |
| --- | --- | --- | --- |
| IgA | 0.079 | 0.016 | 0.228 |
| Passive smoking | 0.066 | 0.022 | 0.228 |
| Anti-mite bedding | 0.161 | 0.027 | 0.228 |
| Damp/mouldy housing | 0.070 | 0.035 | 0.228 |
| Gestational smoking | 0.063 | 0.036 | 0.228 |
| Blood lymphocytes (×10^9^/L) | 0.064 | 0.036 | 0.228 |
| Blood leucocytes (×10^9^/L) | 0.064 | 0.048 | 0.261 |
| Carpets/rugs | 0.083 | 0.109 | 0.470 |
| Age at birth | 0.050 | 0.113 | 0.470 |
| Blood neutrophils (×10^9^/L) | 0.053 | 0.126 | 0.470 |
| Positive BAL virus | 0.053 | 0.136 | 0.470 |
| GERD | 0.051 | 0.186 | 0.570 |
| Allergic rhinoconjunctivitis | 0.044 | 0.195 | 0.570 |
| Age | 0.040 | 0.275 | 0.746 |
| BAL cell number (/mL) | 0.037 | 0.306 | 0.775 |
| RV/TLC | 0.040 | 0.345 | 0.798 |
| Sex | 0.037 | 0.357 | 0.798 |
| Bal lymphocytes (%) | 0.038 | 0.425 | 0.874 |
| LABD > 2/week | 0.042 | 0.437 | 0.874 |
| IgM | 0.036 | 0.497 | 0.875 |
| Cockroaches | 0.082 | 0.502 | 0.875 |
| Cardiopathy | 0.030 | 0.543 | 0.875 |
| Number of severe episodes in the last 12 months | 0.037 | 0.567 | 0.875 |
| Post BD FEF 25% | 0.033 | 0.582 | 0.875 |
| Urban area | 0.030 | 0.582 | 0.875 |
| LABD | 0.029 | 0.664 | 0.875 |
| PPI | 0.028 | 0.677 | 0.875 |
| Pets | 0.028 | 0.678 | 0.875 |
| Blood eosinophils (×10^9^/L) | 0.031 | 0.684 | 0.875 |
| Post BD FEV1 (%) | 0.029 | 0.721 | 0.875 |
| ICS | 0.225 | 0.727 | 0.875 |
| ACT | 0.032 | 0.737 | 0.875 |
| H1 blockers | 0.025 | 0.791 | 0.911 |
| IgG | 0.025 | 0.851 | 0.951 |
| Proven obstructive sleep apnea | 0.026 | 0.894 | 0.971 |
| BAL eosinophils (%) | 0.081 | 0.948 | 0.977 |
| Nasal CS | 0.019 | 0.954 | 0.977 |
| Atopic dermatitis | 0.016 | 0.977 | 0.977 |

*Ig(A/M/G), immunoglobulin; BAL, Bronchoalveolar lavage; GERD, gastroesophageal reflux disease; RV/TLC, residual volume (RV) to total lung capacity (TLC) ratio; LABD, long-acting bronchodilators; Post BD FEF 25% forced expiratory flow measured at 25% of vital capacity after bronchodilator; PPI, proton pump inhibitors; FEV1, forced expiratory volume (1 s); ICS, inhaled corticosteroids; ACT, asthma control test; CS, corticosteroids*
