# Supplementary material for: A microbiome–metabolome signature associated with pediatric severe asthma
Source: PLoS One. 2026 Sep 18;21(9):e0358560. doi: 10.1371/journal.pone.0358560 (PMC13588362; doi:10.1371/journal.pone.0358560)
Supplement: Supplementary file 3 [file pone.0358560.s003.docx]

**S3 Table**

| **Pathway** | **Clinical_Variable** | **Correlation** | **P_Value** |
| --- | --- | --- | --- |
| Arachidonic metabolism | pos_bal_bacter | -0.408 | 0.025 |
| Lysine degradation | pos_bal_bacter | 0.393 | 0.032 |
| Inositol phosphate metabolism | pos_bal_bacter | 0.365 | 0.048 |
