# Supplementary material for: A microbiome–metabolome signature associated with pediatric severe asthma
Source: PLoS One. 2026 Sep 18;21(9):e0358560. doi: 10.1371/journal.pone.0358560 (PMC13588362; doi:10.1371/journal.pone.0358560)
Supplement: Supplementary file 4 [file pone.0358560.s004.docx]

**S4 Table**

| **Metabolite** | **Clinical_Variable** | **Correlation** | **P_Value** | **Pathways_KEGG** |
| --- | --- | --- | --- | --- |
| Phenylacetyl-glutamine | pos_bal_bacter | -0.453 | 0.012 | Phenylalanine metabolism |
| Orotic acid | pos_bal_bacter | 0.446 | 0.014 | Pyrimidine metabolism |
| Creatinine | pos_bal_bacter | -0.438 | 0.016 | Arginine and Proline metabolism |
| Adenosine | pos_bal_bacter | -0.429 | 0.018 | Purine metabolism |
| Aminobutyric acid | Bmi | 0.432 | 0.019 | Arginine and Proline metabolism |
| Picolinic acid | Bmi | 0.431 | 0.019 | Tryptophan metabolism |
| Pro-Gly (prolylglycine) | pos_bal_bacter | 0.415 | 0.023 | Peptides |
| Prostaglandin B1 | pos_bal_bacter | -0.408 | 0.025 | Arachidonic metabolism |
| Metformin | pos_bal_bacter | 0.408 | 0.025 | Unknown |
| 5-Hydroxyindole | Bmi | -0.412 | 0.026 | Tryptophan metabolism |
| Guanosine | pos_bal_bacter | -0.397 | 0.030 | Purine metabolism |
| Threonic acid | Age | 0.395 | 0.031 | Ascorbate and Aldarate metabolism |
| Phenylacetaldehyde | pos_bal_virus | -0.407 | 0.035 | Phenylalanine metabolism |
| 5'-Deoxy-5'-methylthioadenosine | pos_bal_bacter | -0.385 | 0.035 | Cysteine and Methionine |
| Cystine | Age | 0.383 | 0.037 | Cysteine and Methionine |
| N-Acetyl-methionine | pos_bal_virus | 0.396 | 0.041 | Cysteine and Methionine |
| Alpha-Aminoadipic acid | pos_bal_bacter | 0.369 | 0.045 | Lysine degradation |
| Phenyllactic acid | pos_bal_bacter | -0.368 | 0.045 | Phenylalanine metabolism |
| Glyceraldehyde 3-phosphate | pos_bal_bacter | 0.365 | 0.048 | Inositol phosphate metabolism |
