# Supplementary material for: A microbiome–metabolome signature associated with pediatric severe asthma
Source: PLoS One. 2026 Sep 18;21(9):e0358560. doi: 10.1371/journal.pone.0358560 (PMC13588362; doi:10.1371/journal.pone.0358560)
Supplement: Supplementary file 5 [file pone.0358560.s005.docx]

**S5 Table**

| **Metabolites** | **VIP_mean** | **P.values** | **P.adjusted** |
| --- | --- | --- | --- |
| N6-Acetyl-lysine | 1.8055 | 0.007 | 0.1897 |
| Alanyl-proline | 1.7982 | 0.017 | 0.1897 |
| N-acetyl-norvaline | 1.7405 | 0.017 | 0.1897 |
| Lysine | 1.6906 | 0.013 | 0.1897 |
| Val-Pro | 1.6856 | 0.022 | 0.1897 |
| Xanthosine | 1.6540 | 0.031 | 0.1956 |
| Spermine | 1.6287 | 0.011 | 0.1897 |
| Pro-Leu/Leu-Pro | 1.6072 | 0.019 | 0.1897 |
| Homoserine | 1.5522 | 0.008 | 0.1897 |
| Spermidine | 1.4898 | 0.028 | 0.1956 |
| N-Acetyl-ornithine | 1.4749 | 0.022 | 0.1897 |
| Guanosine | 1.4261 | 0.049 | 0.2538 |
| Arginine | 1.3557 | 0.031 | 0.1956 |
| Nicotinamide | 1.3419 | 0.022 | 0.1897 |
| Adenosine | 1.3142 | 0.028 | 0.1956 |
| Pro-Gly | 1.2816 | 0.039 | 0.2159 |
| a-Pinene oxide | 1.2128 | 0.039 | 0.2159 |
