# Supplementary material for: A microbiome–metabolome signature associated with pediatric severe asthma
Source: PLoS One. 2026 Sep 18;21(9):e0358560. doi: 10.1371/journal.pone.0358560 (PMC13588362; doi:10.1371/journal.pone.0358560)
Supplement: Supplementary file 8 [file pone.0358560.s008.docx]

**S3 Fig.**
